# Supplementary material for: Identifying the Most Probable Mammal Reservoir Hosts for Monkeypox Virus Based on Ecological Niche Comparisons
Source: Viruses. 2023 Mar 11;15(3):727. doi: 10.3390/v15030727 (PMC10057484; doi:10.3390/v15030727)
Supplement: Supplementary file 1 [file viruses-15-00727-s001.zip › viruses-2210283-supplementary.pdf]

Table S1: Classification, habitat preference and ecological niche data for all selected mammal species.

Order	Family	Species	African rainforests	Prevalence	Occurrence records (not filtered)	**AUC** (min-max; 95 CI)	D	I	Overlap rank
Eulipotyphla	Erinaceidae	*Atelerix albiventris*	WA	widespread (0.231)	164 (293)	0.861 (0.833-0.888; 0.848-0.873)	0.442	0.733	61
Eulipotyphla	Erinaceidae	*Atelerix frontalis*	not present	narrow-ranged (0.034)	38 (85)	0.874 (0.749-0.922; 0.84-0.908)	0.200	0.478	97
Eulipotyphla	Erinaceidae	*Atelerix sclateri*	not present	narrow-ranged (0.01)	0 (0)	NA	NA	NA	NA
Eulipotyphla	Soricidae	*Crocidura afeworkbekelei*	not present	narrow-ranged (0)	0 (0)	NA	NA	NA	NA
Eulipotyphla	Soricidae	*Crocidura allex*	not present	narrow-ranged (0)	15 (23)	0.949 (0.883-0.984; 0.925-0.972)	0.336	0.657	83
Eulipotyphla	Soricidae	*Crocidura ansellorum*	not present	narrow-ranged (0)	0 (0)	NA	NA	NA	NA
Eulipotyphla	Soricidae	*Crocidura attila*	CA	narrow-ranged (0.027)	3 (5)	NA	NA	NA	NA
Eulipotyphla	Soricidae	*Crocidura baileyi*	not present	narrow-ranged (0.001)	5 (9)	NA	NA	NA	NA
Eulipotyphla	Soricidae	*Crocidura batesi*	CA	narrow-ranged (0.015)	7 (12)	NA	NA	NA	NA
Eulipotyphla	Soricidae	*Crocidura bottegi*	not present	narrow-ranged (0.002)	1 (1)	NA	NA	NA	NA
Eulipotyphla	Soricidae	*Crocidura bottegoides*	not present	narrow-ranged (0)	1 (2)	NA	NA	NA	NA
Eulipotyphla	Soricidae	*Crocidura buettikoferi*	WA	narrow-ranged (0.002)	29 (49)	0.988 (0.944-1; 0.977-0.998)	0.487	0.779	43
Eulipotyphla	Soricidae	*Crocidura caliginea*	CA	narrow-ranged (0.003)	8 (28)	NA	NA	NA	NA
Eulipotyphla	Soricidae	*Crocidura cinderella*	not present	narrow-ranged (0.028)	1 (1)	NA	NA	NA	NA
Eulipotyphla	Soricidae	*Crocidura congobelgica*	CA	narrow-ranged (0.003)	0 (0)	NA	NA	NA	NA
Eulipotyphla	Soricidae	*Crocidura crenata*	CA	narrow-ranged (0.016)	14 (20)	0.697 (0.277-0.975; 0.558-0.835)	0.436	0.776	52
Eulipotyphla	Soricidae	*Crocidura crossei*	WA	narrow-ranged (0.036)	24 (29)	0.967 (0.948-0.985; 0.96-0.975)	0.513	0.806	32
Eulipotyphla	Soricidae	*Crocidura cyanea*	not present	narrow-ranged (0.09)	43 (51)	0.938 (0.887-0.973; 0.921-0.956)	0.289	0.599	89
Eulipotyphla	Soricidae	*Crocidura denti*	CA + WA	narrow-ranged (0.039)	24 (54)	0.93 (0.846-0.982; 0.898-0.962)	0.631	0.8886	13
Eulipotyphla	Soricidae	*Crocidura desperata*	not present	narrow-ranged (0.001)	4 (6)	NA	NA	NA	NA
Eulipotyphla	Soricidae	*Crocidura dolichura*	CA + WA	narrow-ranged (0.049)	41 (66)	0.928 (0.831-0.975; 0.898-0.958)	0.617	0.874	16
Eulipotyphla	Soricidae	*Crocidura douceti*	WA	narrow-ranged (0.003)	11 (15)	NA	NA	NA	NA
Eulipotyphla	Soricidae	*Crocidura eburnea*	WA	narrow-ranged (0.006)	5 (11)	NA	NA	NA	NA
Eulipotyphla	Soricidae	*Crocidura eisentrauti*	WA	narrow-ranged (0)	0 (0)	NA	NA	NA	NA
Eulipotyphla	Soricidae	*Crocidura elgonius*	not present	narrow-ranged (0.003)	16 (33)	0.842 (0.684-0.926; 0.797-0.887)	0.441	0.774	50
Eulipotyphla	Soricidae	*Crocidura erica*	not present	narrow-ranged (0.004)	4 (7)	NA	NA	NA	NA
Eulipotyphla	Soricidae	*Crocidura fingui*	not present	narrow-ranged (0)	0 (0)	NA	NA	NA	NA
Eulipotyphla	Soricidae	*Crocidura fischeri*	not present	narrow-ranged (0)	1 (2)	NA	NA	NA	NA
Eulipotyphla	Soricidae	*Crocidura flavescens*	not present	narrow-ranged (0.01)	18 (35)	0.924 (0.855-0.966; 0.898-0.95)	0.533	0.830	27
Eulipotyphla	Soricidae	*Crocidura foxi*	not present	narrow-ranged (0.055)	16 (20)	0.851 (0.737-0.946; 0.798-0.905)	0.410	0.714	72
Eulipotyphla	Soricidae	*Crocidura fulvastra*	not present	narrow-ranged (0.143)	16 (28)	0.854 (0.64-0.972; 0.787-0.921)	0.486	0.807	37
Eulipotyphla	Soricidae	*Crocidura fumosa*	not present	narrow-ranged (0.001)	20 (29)	0.916 (0.872-0.999; 0.885-0.947)	0.255	0.589	91
Eulipotyphla	Soricidae	*Crocidura fuscomurina*	not present	widespread (0.323)	74 (120)	0.803 (0.712-0.864; 0.77-0.836)	0.474	0.794	41
Eulipotyphla	Soricidae	*Crocidura glassi*	not present	narrow-ranged (0)	3 (9)	NA	NA	NA	NA
Eulipotyphla	Soricidae	*Crocidura goliath*	CA	narrow-ranged (0.033)	25 (34)	0.91 (0.749-0.988; 0.846-0.974)	0.479	0.747	49
Eulipotyphla	Soricidae	*Crocidura grandiceps*	WA	narrow-ranged (0.001)	26 (38)	0.967 (0.921-0.997; 0.949-0.986)	0.465	0.776	46
Eulipotyphla	Soricidae	*Crocidura grassei*	CA	narrow-ranged (0.012)	11 (13)	NA	NA	NA	NA
Eulipotyphla	Soricidae	*Crocidura greenwoodi*	not present	narrow-ranged (0.002)	1 (1)	NA	NA	NA	NA
Eulipotyphla	Soricidae	*Crocidura harenna*	not present	narrow-ranged (0)	0 (0)	NA	NA	NA	NA
Eulipotyphla	Soricidae	*Crocidura hildegardeae*	CA	narrow-ranged (0.077)	67 (135)	0.948 (0.91-0.972; 0.937-0.96)	0.430	0.750	59
Eulipotyphla	Soricidae	*Crocidura hirta*	not present	narrow-ranged (0.133)	144 (206)	0.92 (0.906-0.941; 0.913-0.928)	0.285	0.602	90
Eulipotyphla	Soricidae	*Crocidura jacksoni*	not present	narrow-ranged (0.03)	19 (29)	0.879 (0.836-0.962; 0.855-0.903)	0.506	0.821	30
Eulipotyphla	Soricidae	*Crocidura jouvenetae*	WA	narrow-ranged (0.007)	31 (49)	0.986 (0.961-0.997; 0.98-0.993)	0.506	0.786	38
Eulipotyphla	Soricidae	*Crocidura kivuana*	not present	narrow-ranged (0)	4 (10)	NA	NA	NA	NA
Eulipotyphla	Soricidae	*Crocidura lamottei*	WA	narrow-ranged (0.051)	26 (59)	0.824 (0.727-0.896; 0.784-0.864)	0.432	0.763	56
Eulipotyphla	Soricidae	*Crocidura lanosa*	not present	narrow-ranged (0)	6 (10)	NA	NA	NA	NA
Eulipotyphla	Soricidae	*Crocidura latona*	CA	narrow-ranged (0.005)	7 (8)	NA	NA	NA	NA
Eulipotyphla	Soricidae	*Crocidura littoralis*	CA	narrow-ranged (0.062)	19 (45)	0.953 (0.773-0.99; 0.91-0.996)	0.629	0.887	14
Eulipotyphla	Soricidae	*Crocidura longipes*	not present	narrow-ranged (0)	1 (1)	NA	NA	NA	NA
Eulipotyphla	Soricidae	*Crocidura lucina*	not present	narrow-ranged (0)	2 (4)	NA	NA	NA	NA
Eulipotyphla	Soricidae	*Crocidura ludia*	CA	narrow-ranged (0.016)	15 (33)	0.873 (0.389-0.974; 0.755-0.991)	0.543	0.837	23
Eulipotyphla	Soricidae	*Crocidura luna*	not present	narrow-ranged (0.06)	79 (126)	0.928 (0.9-0.953; 0.917-0.939)	0.424	0.754	60
Eulipotyphla	Soricidae	*Crocidura lusitania*	not present	narrow-ranged (0.067)	3 (3)	NA	NA	NA	NA
Eulipotyphla	Soricidae	*Crocidura lwiroensis*	not present	narrow-ranged (0)	2 (2)	NA	NA	NA	NA
Eulipotyphla	Soricidae	*Crocidura macarthuri*	not present	narrow-ranged (0.015)	2 (2)	NA	NA	NA	NA
Eulipotyphla	Soricidae	*Crocidura macmillani*	not present	narrow-ranged (0.001)	1 (1)	NA	NA	NA	NA
Eulipotyphla	Soricidae	*Crocidura macowi*	not present	narrow-ranged (0)	0 (0)	NA	NA	NA	NA
Eulipotyphla	Soricidae	*Crocidura manengubae*	WA	narrow-ranged (0)	0 (0)	NA	NA	NA	NA
Eulipotyphla	Soricidae	*Crocidura maquassiensis*	not present	narrow-ranged (0.011)	1 (1)	NA	NA	NA	NA
Eulipotyphla	Soricidae	*Crocidura mariquensis*	not present	narrow-ranged (0.068)	28 (40)	0.92 (0.867-0.963; 0.9-0.941)	0.319	0.656	84
Eulipotyphla	Soricidae	*Crocidura maurisca*	CA	narrow-ranged (0.019)	15 (18)	0.861 (0.765-0.996; 0.801-0.922)	0.402	0.743	68
Eulipotyphla	Soricidae	*Crocidura mdumai*	not present	narrow-ranged (0)	1 (2)	NA	NA	NA	NA
Eulipotyphla	Soricidae	*Crocidura monax*	not present	narrow-ranged (0)	5 (8)	NA	NA	NA	NA
Eulipotyphla	Soricidae	*Crocidura montis*	not present	narrow-ranged (0.001)	13 (28)	NA	NA	NA	NA
Eulipotyphla	Soricidae	*Crocidura munissii*	not present	narrow-ranged (0.001)	7 (18)	NA	NA	NA	NA
Eulipotyphla	Soricidae	*Crocidura muricauda*	WA	narrow-ranged (0.013)	24 (48)	0.991 (0.982-0.997; 0.988-0.994)	0.533	0.834	26
Eulipotyphla	Soricidae	*Crocidura mutesae*	CA	narrow-ranged (0.001)	4 (4)	NA	NA	NA	NA
Eulipotyphla	Soricidae	*Crocidura nana*	not present	narrow-ranged (0)	1 (2)	NA	NA	NA	NA
Eulipotyphla	Soricidae	*Crocidura nanilla*	not present	narrow-ranged (0.025)	3 (4)	NA	NA	NA	NA
Eulipotyphla	Soricidae	*Crocidura newmarki*	not present	narrow-ranged (0)	1 (5)	NA	NA	NA	NA
Eulipotyphla	Soricidae	*Crocidura nigeriae*	WA	narrow-ranged (0.007)	13 (14)	NA	NA	NA	NA
Eulipotyphla	Soricidae	*Crocidura nigricans*	not present	narrow-ranged (0.004)	0 (0)	NA	NA	NA	NA
Eulipotyphla	Soricidae	*Crocidura nigrofusca*	not present	narrow-ranged (0.125)	32 (54)	0.859 (0.752-0.932; 0.819-0.9)	0.367	0.695	79
Eulipotyphla	Soricidae	*Crocidura nimbae*	WA	narrow-ranged (0.001)	8 (9)	NA	NA	NA	NA

Eulipotyphla	Soricidae	*Crocidura nimbasilvanus*	WA	narrow-ranged (0.002)	6 (7)	NA	NA	NA	NA
Eulipotyphla	Soricidae	*Crocidura niobe*	not present	narrow-ranged (0.001)	11 (25)	NA	NA	NA	NA
Eulipotyphla	Soricidae	*Crocidura obscurior*	WA	narrow-ranged (0.012)	28 (68)	0.995 (0.983-1; 0.992-0.998)	0.489	0.789	39
Eulipotyphla	Soricidae	*Crocidura olivieri*	CA + WA	widespread (0.521)	421 (790)	0.897 (0.876-0.915; 0.89-0.904)	0.654	0.908	9
Eulipotyphla	Soricidae	*Crocidura parvipes*	CA	narrow-ranged (0.184)	17 (36)	0.793 (0.587-0.98; 0.704-0.882)	0.408	0.745	67
Eulipotyphla	Soricidae	*Crocidura pasha*	not present	narrow-ranged (0.018)	0 (0)	NA	NA	NA	NA
Eulipotyphla	Soricidae	*Crocidura phaeura*	not present	narrow-ranged (0)	1 (2)	NA	NA	NA	NA
Eulipotyphla	Soricidae	*Crocidura picea*	WA	narrow-ranged (0)	0 (0)	NA	NA	NA	NA
Eulipotyphla	Soricidae	*Crocidura pitmani*	not present	narrow-ranged (0.001)	0 (0)	NA	NA	NA	NA
Eulipotyphla	Soricidae	*Crocidura planiceps*	not present	narrow-ranged (0)	1 (1)	NA	NA	NA	NA
Eulipotyphla	Soricidae	*Crocidura poensis*	WA	narrow-ranged (0.036)	54 (79)	0.977 (0.963-0.985; 0.973-0.982)	0.500	0.797	36
Eulipotyphla	Soricidae	*Crocidura polia*	CA	narrow-ranged (0)	0 (0)	NA	NA	NA	NA
Eulipotyphla	Soricidae	*Crocidura raineyi*	not present	narrow-ranged (0)	1 (1)	NA	NA	NA	NA
Eulipotyphla	Soricidae	*Crocidura roosevelti*	not present	narrow-ranged (0.065)	0 (0)	NA	NA	NA	NA
Eulipotyphla	Soricidae	*Crocidura selina*	not present	narrow-ranged (0.001)	3 (5)	NA	NA	NA	NA
Eulipotyphla	Soricidae	*Crocidura silacea*	not present	narrow-ranged (0.011)	6 (6)	NA	NA	NA	NA
Eulipotyphla	Soricidae	*Crocidura smithii*	not present	narrow-ranged (0.006)	0 (0)	NA	NA	NA	NA
Eulipotyphla	Soricidae	*Crocidura somalica*	not present	narrow-ranged (0.006)	0 (0)	NA	NA	NA	NA
Eulipotyphla	Soricidae	*Crocidura stenocephala*	not present	narrow-ranged (0)	3 (6)	NA	NA	NA	NA
Eulipotyphla	Soricidae	*Crocidura tansaniana*	not present	narrow-ranged (0)	2 (10)	NA	NA	NA	NA
Eulipotyphla	Soricidae	*Crocidura tarella*	not present	narrow-ranged (0)	1 (1)	NA	NA	NA	NA
Eulipotyphla	Soricidae	*Crocidura tarfayensis*	not present	narrow-ranged (0.009)	5 (5)	NA	NA	NA	NA
Eulipotyphla	Soricidae	*Crocidura telfordi*	not present	narrow-ranged (0)	4 (11)	NA	NA	NA	NA
Eulipotyphla	Soricidae	*Crocidura thalia*	not present	narrow-ranged (0.005)	9 (21)	NA	NA	NA	NA
Eulipotyphla	Soricidae	*Crocidura theresae*	WA	narrow-ranged (0.009)	40 (64)	0.966 (0.95-0.992; 0.957-0.975)	0.652	0.907	10
Eulipotyphla	Soricidae	*Crocidura thomensis*	not present	narrow-ranged (0)	4 (4)	NA	NA	NA	NA
Eulipotyphla	Soricidae	*Crocidura turba*	CA	narrow-ranged (0.16)	64 (118)	0.855 (0.799-0.931; 0.83-0.88)	0.437	0.763	54
Eulipotyphla	Soricidae	*Crocidura ultima*	not present	narrow-ranged (0)	0 (0)	NA	NA	NA	NA
Eulipotyphla	Soricidae	*Crocidura usambarae*	not present	narrow-ranged (0)	6 (13)	NA	NA	NA	NA
Eulipotyphla	Soricidae	*Crocidura viaria*	not present	widespread (0.27)	17 (20)	NA	NA	NA	NA
Eulipotyphla	Soricidae	*Crocidura virgata*	WA	narrow-ranged (0)	0 (0)	NA	NA	NA	NA
Eulipotyphla	Soricidae	*Crocidura voi*	not present	narrow-ranged (0.099)	2 (3)	NA	NA	NA	NA
Eulipotyphla	Soricidae	*Crocidura wimmeri*	WA	narrow-ranged (0)	3 (3)	NA	NA	NA	NA
Eulipotyphla	Soricidae	*Crocidura xantippe*	not present	narrow-ranged (0.001)	2 (3)	NA	NA	NA	NA
Eulipotyphla	Soricidae	*Crocidura yaldeni*	not present	narrow-ranged (0)	1 (1)	NA	NA	NA	NA
Eulipotyphla	Soricidae	*Crocidura yankariensis*	not present	narrow-ranged (0.046)	1 (1)	NA	NA	NA	NA
Eulipotyphla	Soricidae	*Crocidura zaphiri*	not present	narrow-ranged (0)	0 (0)	NA	NA	NA	NA
Eulipotyphla	Soricidae	*Crocidura zimmeri*	not present	narrow-ranged (0)	1 (2)	NA	NA	NA	NA
Macroscelidea	Macroscelididae	*Petrodromus tetradactylus*	CA	narrow-ranged (0.103)	88 (170)	0.925 (0.908-0.945; 0.917-0.934)	0.382	0.706	75
Primate	Cercopithecidae	*Allenopithecus nigroviridis*	CA	narrow-ranged (0.005)	5 (8)	NA	NA	NA	NA
Primate	Cercopithecidae	*Cercocebus agilis*	CA	narrow-ranged (0.033)	18 (42)	0.927 (0.824-0.981; 0.897-0.957)	0.563	0.857	21
Primate	Cercopithecidae	*Cercocebus atys*	WA	narrow-ranged (0.01)	28 (81)	0.959 (0.916-0.991; 0.945-0.974)	0.437	0.754	58
Primate	Cercopithecidae	*Cercocebus chrysogaster*	CA	narrow-ranged (0.003)	1 (1)	NA	NA	NA	NA
Primate	Cercopithecidae	*Cercocebus galeritus*	not present	narrow-ranged (0)	4 (28)	NA	NA	NA	NA
Primate	Cercopithecidae	*Cercocebus lunulatus*	WA	narrow-ranged (0.005)	3 (5)	NA	NA	NA	NA
Primate	Cercopithecidae	*Cercocebus sanjei*	not present	narrow-ranged (0)	5 (12)	NA	NA	NA	NA
Primate	Cercopithecidae	*Cercocebus torquatus*	CA + WA	narrow-ranged (0.009)	32 (51)	0.978 (0.961-0.993; 0.972-0.984)	0.427	0.736	64
Primate	Cercopithecidae	*Cercopithecus ascanius*	CA	narrow-ranged (0.086)	149 (688)	0.956 (0.929-0.976; 0.947-0.965)	0.653	0.897	11
Primate	Cercopithecidae	*Cercopithecus campbelli*	WA	narrow-ranged (0.01)	50 (89)	0.989 (0.982-0.996; 0.986-0.992)	0.531	0.791	31
Primate	Cercopithecidae	*Cercopithecus cephus*	CA	narrow-ranged (0.027)	63 (123)	0.986 (0.981-0.994; 0.984-0.989)	0.424	0.683	74
Primate	Cercopithecidae	*Cercopithecus denti*	CA	narrow-ranged (0.013)	13 (25)	NA	NA	NA	NA
Primate	Cercopithecidae	*Cercopithecus diana*	WA	narrow-ranged (0.007)	40 (153)	0.988 (0.941-0.997; 0.977-0.998)	0.502	0.807	35
Primate	Cercopithecidae	*Cercopithecus dryas*	CA	narrow-ranged (0)	4 (8)	NA	NA	NA	NA
Primate	Cercopithecidae	*Cercopithecus erythrogaster*	WA	narrow-ranged (0.002)	33 (69)	0.99 (0.971-0.997; 0.984-0.995)	0.411	0.733	69
Primate	Cercopithecidae	*Cercopithecus erythrotis*	CA + WA	narrow-ranged (0.002)	20 (34)	0.994 (0.984-0.999; 0.991-0.996)	0.417	0.723	71
Primate	Cercopithecidae	*Cercopithecus hamlyni*	CA	narrow-ranged (0.007)	8 (8)	NA	NA	NA	NA
Primate	Cercopithecidae	*Cercopithecus lomamiensis*	CA	narrow-ranged (0.001)	3 (6)	NA	NA	NA	NA
Primate	Cercopithecidae	*Cercopithecus lowei*	WA	narrow-ranged (0.009)	36 (89)	0.976 (0.869-0.996; 0.953-1)	0.361	0.655	82
Primate	Cercopithecidae	*Cercopithecus mitis*	CA	narrow-ranged (0.088)	453 (2228)	0.951 (0.943-0.958; 0.947-0.954)	0.510	0.803	34
Primate	Cercopithecidae	*Cercopithecus mona*	WA	narrow-ranged (0.021)	98 (201)	0.988 (0.982-0.995; 0.986-0.99)	0.384	0.689	77
Primate	Cercopithecidae	*Cercopithecus neglectus*	CA	narrow-ranged (0.075)	48 (110)	0.927 (0.856-0.967; 0.904-0.95)	0.641	0.890	12
Primate	Cercopithecidae	*Cercopithecus nictitans*	CA + WA	narrow-ranged (0.044)	121 (210)	0.974 (0.964-0.983; 0.97-0.978)	0.630	0.852	18
Primate	Cercopithecidae	*Cercopithecus petaurista*	WA	narrow-ranged (0.018)	77 (111)	0.982 (0.963-0.991; 0.977-0.987)	0.588	0.831	22
Primate	Cercopithecidae	*Cercopithecus pogonias*	CA + WA	narrow-ranged (0.036)	48 (70)	0.972 (0.958-0.981; 0.967-0.977)	0.563	0.807	25
Primate	Cercopithecidae	*Cercopithecus roloway*	WA	narrow-ranged (0)	5 (5)	NA	NA	NA	NA
Primate	Cercopithecidae	*Cercopithecus sclateri*	WA	narrow-ranged (0.001)	8 (13)	NA	NA	NA	NA
Primate	Cercopithecidae	*Cercopithecus wolfi*	CA	narrow-ranged (0.023)	24 (35)	0.956 (0.736-0.993; 0.907-1)	0.472	0.795	42
Primate	Cercopithecidae	*Chlorocebus aethiops*	not present	narrow-ranged (0.038)	75 (264)	0.943 (0.915-0.969; 0.932-0.953)	0.373	0.701	76
Primate	Cercopithecidae	*Chlorocebus cynosuros*	CA	narrow-ranged (0.101)	56 (157)	0.928 (0.868-0.974; 0.908-0.948)	0.253	0.563	93
Primate	Cercopithecidae	*Chlorocebus djamdjamensis*	not present	narrow-ranged (0)	10 (33)	NA	NA	NA	NA
Primate	Cercopithecidae	*Chlorocebus pygerythrus*	not present	narrow-ranged (0.153)	990 (6184)	0.925 (0.918-0.93; 0.922-0.928)	0.373	0.693	78
Primate	Cercopithecidae	*Chlorocebus sabaeus*	WA	narrow-ranged (0.047)	84 (729)	0.975 (0.96-0.986; 0.97-0.98)	0.310	0.617	87
Primate	Cercopithecidae	*Chlorocebus tantalus*	CA + WA	narrow-ranged (0.13)	143 (560)	0.906 (0.823-0.946; 0.882-0.93)	0.450	0.764	51
Primate	Cercopithecidae	*Piliocolobus badius*	WA	narrow-ranged (0.011)	70 (261)	0.986 (0.976-0.993; 0.983-0.989)	0.434	0.729	63
Primate	Cercopithecidae	*Piliocolobus bouvieri*	CA	narrow-ranged (0.001)	1 (1)	NA	NA	NA	NA
Primate	Cercopithecidae	*Piliocolobus epieni*	WA	narrow-ranged (0)	0 (0)	NA	NA	NA	NA
Primate	Cercopithecidae	*Piliocolobus foai*	not present	narrow-ranged (0)	1 (1)	NA	NA	NA	NA
Primate	Cercopithecidae	*Piliocolobus gordonorum*	not present	narrow-ranged (0)	10 (27)	NA	NA	NA	NA

Primate	Cercopithecidae	*Piliocolobus kirkii*	not present	narrow-ranged (0)	15 (318)	NA	NA	NA	NA
Primate	Cercopithecidae	*Piliocolobus langi*	CA	narrow-ranged (0.002)	1 (1)	NA	NA	NA	NA
Primate	Cercopithecidae	*Piliocolobus lulindicus*	CA	narrow-ranged (0.003)	0 (0)	NA	NA	NA	NA
Primate	Cercopithecidae	*Piliocolobus oustaleti*	CA	narrow-ranged (0.014)	10 (14)	NA	NA	NA	NA
Primate	Cercopithecidae	*Piliocolobus parmentieri*	CA	narrow-ranged (0.001)	2 (3)	NA	NA	NA	NA
Primate	Cercopithecidae	*Piliocolobus pennantii*	not present	narrow-ranged (0)	2 (5)	NA	NA	NA	NA
Primate	Cercopithecidae	*Piliocolobus preussi*	WA	narrow-ranged (0)	1 (1)	NA	NA	NA	NA
Primate	Cercopithecidae	*Piliocolobus rufomitratus*	not present	narrow-ranged (0)	6 (22)	NA	NA	NA	NA
Primate	Cercopithecidae	*Piliocolobus semlikiensis*	CA	narrow-ranged (0.002)	3 (3)	NA	NA	NA	NA
Primate	Cercopithecidae	*Piliocolobus tephrosceles*	not present	narrow-ranged (0)	27 (181)	0.885 (0.62-0.997; 0.8-0.969)	0.355	0.687	81
Primate	Cercopithecidae	*Piliocolobus tholloni*	CA	narrow-ranged (0.016)	8 (15)	NA	NA	NA	NA
Primate	Hominidae	*Pan paniscus*	CA	narrow-ranged (0.014)	8 (8)	NA	NA	NA	NA
Primate	Hominidae	*Pan troglodytes*	CA + WA	narrow-ranged (0.076)	205 (1302)	0.948 (0.93-0.96; 0.943-0.954)	0.669	0.912	7
Primate	Lorisidae	*Perodicticus edwardsi*	CA + WA	narrow-ranged (0.08)	41 (55)	0.968 (0.959-0.977; 0.964-0.972)	0.605	0.844	19
Primate	Lorisidae	*Perodicticus ibeanus*	CA	narrow-ranged (0.028)	24 (36)	0.818 (0.686-0.951; 0.761-0.875)	0.407	0.732	70
Primate	Lorisidae	*Perodicticus potto*	WA	narrow-ranged (0.022)	53 (90)	0.983 (0.978-0.988; 0.98-0.985)	0.431	0.726	66
Rodentia	Dipodidae	*Jaculus jaculus*	not present	widespread (0.267)	297 (587)	0.922 (0.908-0.934; 0.916-0.927)	0.127	0.364	98
Rodentia	Gliridae	*Graphiurus angolensis*	not present	narrow-ranged (0.007)	0 (0)	NA	NA	NA	NA
Rodentia	Gliridae	*Graphiurus christyi*	CA	narrow-ranged (0.013)	28 (31)	0.954 (0.881-0.992; 0.929-0.979)	0.479	0.792	40
Rodentia	Gliridae	*Graphiurus crassicaudatus*	WA	narrow-ranged (0.017)	32 (43)	0.974 (0.926-0.997; 0.958-0.99)	0.423	0.744	62
Rodentia	Gliridae	*Graphiurus johnstoni*	not present	narrow-ranged (0)	0 (0)	NA	NA	NA	NA
Rodentia	Gliridae	*Graphiurus kelleni*	WA	narrow-ranged (0.091)	28 (36)	0.802 (0.734-0.898; 0.77-0.833)	0.500	0.814	33
Rodentia	Gliridae	*Graphiurus lorraineus*	CA + WA	narrow-ranged (0.114)	47 (51)	0.926 (0.888-0.955; 0.913-0.939)	0.727	0.917	2
Rodentia	Gliridae	*Graphiurus microtis*	not present	narrow-ranged (0.109)	52 (76)	0.874 (0.758-0.928; 0.84-0.908)	0.363	0.685	80
Rodentia	Gliridae	*Graphiurus monardi*	not present	narrow-ranged (0.001)	0 (0)	NA	NA	NA	NA
Rodentia	Gliridae	*Graphiurus murinus*	not present	narrow-ranged (0.032)	163 (319)	0.959 (0.931-0.977; 0.95-0.967)	0.390	0.720	73
Rodentia	Gliridae	*Graphiurus nagtglasii*	CA + WA	narrow-ranged (0.029)	40 (52)	0.976 (0.945-0.991; 0.968-0.984)	0.540	0.822	28
Rodentia	Gliridae	*Graphiurus ocularis*	not present	narrow-ranged (0.011)	17 (29)	0.995 (0.978-1; 0.991-0.999)	0.127	0.302	99
Rodentia	Gliridae	*Graphiurus platyops*	not present	narrow-ranged (0.011)	1 (1)	NA	NA	NA	NA
Rodentia	Gliridae	*Graphiurus rupicola*	not present	narrow-ranged (0.002)	1 (1)	NA	NA	NA	NA
Rodentia	Gliridae	*Graphiurus surdus*	CA	narrow-ranged (0.002)	1 (1)	NA	NA	NA	NA
Rodentia	Gliridae	*Graphiurus walterverheyeni*	CA	narrow-ranged (0.003)	0 (0)	NA	NA	NA	NA
Rodentia	Muridae	*Malacomys cansdalei*	WA	narrow-ranged (0.006)	17 (32)	0.954 (0.903-0.979; 0.939-0.969)	0.474	0.791	44
Rodentia	Muridae	*Malacomys edwardsi*	WA	narrow-ranged (0.016)	72 (120)	0.988 (0.976-0.993; 0.985-0.991)	0.615	0.886	15
Rodentia	Muridae	*Malacomys longipes*	CA + WA	narrow-ranged (0.04)	127 (196)	0.965 (0.937-0.975; 0.958-0.971)	0.685	0.902	6
Rodentia	Muridae	*Oenomys hypoxanthus*	CA + WA	narrow-ranged (0.137)	248 (395)	0.944 (0.927-0.958; 0.938-0.95)	0.668	0.904	8
Rodentia	Muridae	*Oenomys ornatus*	WA	narrow-ranged (0.005)	5 (7)	NA	NA	NA	NA
Rodentia	Muridae	*Stochomys longicaudatus*	CA + WA	narrow-ranged (0.061)	122 (162)	0.957 (0.939-0.971; 0.951-0.963)	0.698	0.921	5
Rodentia	Nesomyidae	*Cricetomys ansorgei*	CA	narrow-ranged (0.188)	94 (132)	0.926 (0.908-0.953; 0.917-0.934)	0.470	0.766	47
Rodentia	Nesomyidae	*Cricetomys emini*	CA + WA	narrow-ranged (0.123)	134 (193)	0.964 (0.952-0.974; 0.96-0.968)	0.627	0.864	17
Rodentia	Nesomyidae	*Cricetomys gambianus*	WA	narrow-ranged (0.136)	229 (533)	0.941 (0.925-0.953; 0.935-0.947)	0.564	0.856	20
Rodentia	Sciuridae	*Funisciurus anerythrus*	CA + WA	narrow-ranged (0.075)	112 (142)	0.952 (0.93-0.973; 0.945-0.96)	0.746	0.931	1
Rodentia	Sciuridae	*Funisciurus bayonii*	not present	narrow-ranged (0.013)	9 (13)	NA	NA	NA	NA
Rodentia	Sciuridae	*Funisciurus carruthersi*	not present	narrow-ranged (0.003)	53 (106)	0.967 (0.93-0.997; 0.955-0.98)	0.300	0.633	86
Rodentia	Sciuridae	*Funisciurus congicus*	CA	narrow-ranged (0.057)	92 (152)	0.884 (0.843-0.91; 0.871-0.898)	0.516	0.824	29
Rodentia	Sciuridae	*Funisciurus duchaillui*	CA	narrow-ranged (0)	0 (0)	NA	NA	NA	NA
Rodentia	Sciuridae	*Funisciurus isabella*	CA + WA	narrow-ranged (0.019)	24 (42)	0.952 (0.914-0.983; 0.936-0.967)	0.541	0.837	24
Rodentia	Sciuridae	*Funisciurus lemniscatus*	CA	narrow-ranged (0.02)	37 (52)	0.976 (0.943-0.989; 0.968-0.984)	0.458	0.735	57
Rodentia	Sciuridae	*Funisciurus leucogenys*	CA + WA	narrow-ranged (0.014)	49 (93)	0.983 (0.946-0.993; 0.975-0.992)	0.487	0.765	45
Rodentia	Sciuridae	*Funisciurus pyrropus*	CA + WA	narrow-ranged (0.071)	159 (207)	0.95 (0.925-0.973; 0.941-0.958)	0.719	0.918	3
Rodentia	Sciuridae	*Funisciurus substriatus*	WA	narrow-ranged (0.007)	28 (56)	0.925 (0.852-0.989; 0.893-0.957)	0.438	0.759	55
Rodentia	Sciuridae	*Heliosciurus gambianus*	WA	narrow-ranged (0.155)	212 (549)	0.898 (0.884-0.911; 0.893-0.902)	0.430	0.728	65
Rodentia	Sciuridae	*Heliosciurus mutabilis*	not present	narrow-ranged (0.035)	52 (122)	0.938 (0.809-0.993; 0.896-0.98)	0.214	0.510	95
Rodentia	Sciuridae	*Heliosciurus punctatus*	WA	narrow-ranged (0.011)	26 (43)	0.994 (0.984-0.998; 0.991-0.996)	0.465	0.762	48
Rodentia	Sciuridae	*Heliosciurus rufobrachium*	CA + WA	narrow-ranged (0.097)	306 (469)	0.948 (0.938-0.961; 0.943-0.952)	0.705	0.915	4
Rodentia	Sciuridae	*Heliosciurus ruwenzorii*	not present	narrow-ranged (0.005)	47 (89)	0.928 (0.828-0.979; 0.899-0.957)	0.318	0.654	85
Rodentia	Sciuridae	*Heliosciurus undulatus*	not present	narrow-ranged (0.004)	28 (112)	0.995 (0.987-0.998; 0.993-0.997)	0.300	0.591	88
Rodentia	Sciuridae	*Xerus erythropus*	CA + WA	widespread (0.29)	413 (771)	0.911 (0.895-0.924; 0.905-0.917)	0.456	0.754	53
Rodentia	Sciuridae	*Xerus inauris*	not present	narrow-ranged (0.05)	350 (842)	0.968 (0.957-0.975; 0.964-0.972)	0.203	0.484	96
Rodentia	Sciuridae	*Xerus princeps*	not present	narrow-ranged (0.008)	23 (39)	0.908 (0.856-0.987; 0.884-0.933)	0.266	0.577	92
Rodentia	Sciuridae	*Xerus rutilus*	not present	narrow-ranged (0.051)	141 (332)	0.971 (0.952-0.984; 0.964-0.978)	0.250	0.524	94

Abbreviations: AUC: area under the curve; CI: Confidence interval; D: Schoener's D; I: Hellinger's I; CA: Central African rainforests; WA: West African rainforests; NA: not applicable

Table S2: Geographic coordinates of the 109 occurrence records used for the MPXV niche.

Year	Human/Animal	Location	Country	Latitude	Longitude
1987	Human	70km of Lambarené	Gabon	-0.31	10.46
2016	Human	Rehou 4	CAR	5.47	21.84
1970	Human	Bokenda	DRC	0.96	20.22
1970	Human	Boudua	Liberia	6.19	-7.98
1970	Human	Taa	Liberia	6.25	-8.03
1970	Human	Aguebu	Sierra Leone	8.20	-12.40
1971	Human	Bossematié (Bosmatché)	Côte d'Ivoire	6.61	-3.44
1971	Human	Ihie Umuduru	Nigeria	5.02	7.31
1972	Human	Libela	DRC	2.63	22.19
1972	Human	Bokokolo (Bokokole)	DRC	0.70	22.42
1973	Human	Bogon	DRC	2.91	19.69
1973	Human	Bombana	DRC	2.77	21.81
1974	Human	Bumba	DRC	2.19	22.47
1975	Human	Iba	DRC	-2.43	17.78
1975	Human	Djungula	DRC	-3.27	22.39
1976	Human	Ebata	DRC	2.50	21.74
1976	Human	Yamgomba	DRC	2.34	22.30
1976	Human	Masina	DRC	-5.86	17.05
1977	Human	Yamagbe-Bohumbe	DRC	2.61	21.85
1977	Human	Iwadji	DRC	-3.13	22.20
1977	Human	Bwalayulu	DRC	-4.52	17.99
1977	Human	Selebo II (Katanti)	DRC	-2.27	27.10
1978	Human	Mwanzita	DRC	-7.10	17.89
1978	Human	Ikela	DRC	-1.05	23.36
1978	Human	Mongo Senge	DRC	-3.40	22.37
1978	Human	Imbimbi	DRC	-4.70	19.36
1978	Human	Okela	DRC	-3.64	22.18
1978	Human	Ekodji	DRC	-3.59	22.22
1978	Human	Mindembo	DRC	2.44	21.98
1978	Human	Yaliengo	DRC	2.38	21.89
1978	Human	Apoko	DRC	-3.32	22.64
1978	Human	Omifunfun	Nigeria	7.16	4.60
1979	Human	Ekidmékoé	Cameroon	3.63	11.65
1979	Human	Bosokuma	DRC	1.81	20.60
1979	Human	Olonga	DRC	-3.90	22.76
1980	Human	Moloundou	Cameroon	2.03	15.22

1981	Human	Gbétitapéa	Côte d'Ivoire	6.80	-6.46
1982	Human	Kikongo (Camp Bulumbu III)	DRC	-4.98	18.77
1982	Human	Kibwe	DRC	-2.22	27.12
1983	Human	Iyeke	DRC	-4.00	21.30
1983	Human	Idumbe	DRC	-3.93	21.55
1984	Human	Lidjombo	CAR	2.70	16.10
1985	Animal	Bodjoki (40 km of Yambuku)	DRC	2.98	22.30
1986	Human	Yandongi	DRC	2.84	22.27
1986	Human	Ikubi	DRC	-4.55	19.56
1989	Human	Ekoum-Douma	Cameroon	4.17	11.53
1996	Human	Akungula	DRC	-3.26	23.98
1996	Human	Ekanga	DRC	-3.34	24.21
1997	Human	Ombeka	DRC	-3.40	24.05
2001	Human	Abuzi	DRC	3.69	22.15
2001	Human	Boso Mane	DRC	1.76	20.05
2001	Human	En Dongale	DRC	1.77	20.55
2003	Human	Doungou	Republic of Congo	2.05	18.05
2008	Human	Boende	DRC	-0.28	20.88
2010	Human	Mongoumba	CAR	3.64	18.59
2010	Human	Boyele Port	Republic of Congo	2.55	18.18
2010	Human	Ebobo	Republic of Congo	3.17	18.48
2011	Human	Boboro (Walikale)	DRC	-1.33	27.97
2012	Human	Mabaka (Shabunda)	DRC	-2.93	27.13
2013	Human	Bokungu	DRC	-0.60	22.32
2014	Animal	Sanaga-Yong	Cameroon	4.87	13.17
2014	Human	Kpetema	Sierra Leone	7.75	-11.63
2015	Human	Lengo	CAR	5.68	22.87
2015	Human	Bria/Koupou 2	CAR	6.75	22.00
2016	Animal	Mefou Primate Sanctuary	Cameroon	3.62	11.58
2016	Human	Bandoufou	CAR	4.90	22.94
2016	Human	Fadama	CAR	5.73	22.76
2016	Human	Bogbengo, Aketi	DRC	2.93	23.74
2017	Human	Bao	CAR	4.44	22.35
2017	Human	Café-Machado	CAR	3.83	18.04
2017	Animal	Taï National Park	Côte d'Ivoire	5.80	-7.32
2017	Animal	Taï National Park	Côte d'Ivoire	5.88	-7.35
2017	Human	Agbura	Nigeria	4.85	6.27
2017	Human	Moualé	Republic of Congo	3.38	17.18

2017	Human	Manfoueté	Republic of Congo	2.36	17.66
2017	Human	Kpaku	Sierra Leone	7.37	-11.63
2017	Human	Tenken	Liberia	5.01	-7.86
2018	Human	Bokoka/Bagandou	CAR	3.90	17.92
2018	Human	Bakouma	CAR	5.71	22.78
2018	Human	Mbalazimé (Bangassou)	CAR	4.81	22.87
2018	Human	Rafaï	CAR	4.98	23.94
2018	Human	Zoméa	CAR	3.65	18.02
2018	Human	Bangassou	CAR	5.01	23.15
2018	Human	Bossembele	CAR	5.27	17.63
2018	Animal	Taï National Park	Côte d'Ivoire	5.82	-7.27
2019	Human	Tomba 1, Ayos Health District	Cameroon	4.27	12.55
2019	Human	Ouadjimi/Bambari	CAR	5.74	21.05
2019	Human	Boda/Géomètre	CAR	4.31	17.47
2019	Human	Loko	CAR	3.64	18.06
2019	Human	Ndolobo	CAR	3.82	17.85
2019	Human	Bomango/Boda	CAR	4.06	17.47
2019	Human	Moloukou	CAR	3.74	17.52
2019	Human	Ngoto	CAR	4.02	17.34
2019	Human	Moboma	CAR	3.73	17.84
2019	Human	Makotipoko, Gambona	DRC	-2.00	16.32
2020	Human	Bambio / Bounguele	CAR	3.94	16.97
2020	Human	Wodo/Nola	CAR	3.52	16.05
2020	Human	Mokongo/Bagandou	CAR	3.93	18.27
2020	Human	Combattant	CAR	5.01	23.15
2020	Human	Kembé (Café Bangui)	CAR	4.62	21.89
2021	Human	Bagawa (Bria)	CAR	6.75	22.00
2021	Human	Gbodo/Bangassou	CAR	4.32	22.55
2021	Human	Massangai/ Bambio	CAR	3.94	16.97
2021	Human	Moloukou/Dakota	CAR	3.74	17.52
2021	Human	SCED Ndelengue	CAR	3.44	16.39
2021	Human	Grima	CAR	4.08	17.07
2021	Human	Bania	CAR	4.60	16.10
2021	Human	Nazembe	CAR	4.16	15.96
2021	Human	Balego/Bayanga	CAR	2.91	16.27

Abbreviations: CAR: Central African Republic; DRC: Democratic Republic of the Congo.

Figure S1: Ecological niches of mammal species ranked between the 11th and 99th positions for their overlap with the MPXV niche.

Black circles indicate localities used to build the distribution model. The probabilities of occurrence are highlighted using different colours: blue grey for probabilities < 0.5; turquoise green for 0.5 < p < 0.75; yellowish green for 0.75 < p < 0.9; and yellow for p > 0.9. The red line is the IUCN distribution of the species [60]. At the left of the maps are indicated the number of occurrence records (n) used to infer the ecological niche, the Schoener's D and Hellinger's I values summarizing niche overlap between mammal species and MPXV, and the rank based on overlap analyses with the MPXV niche.

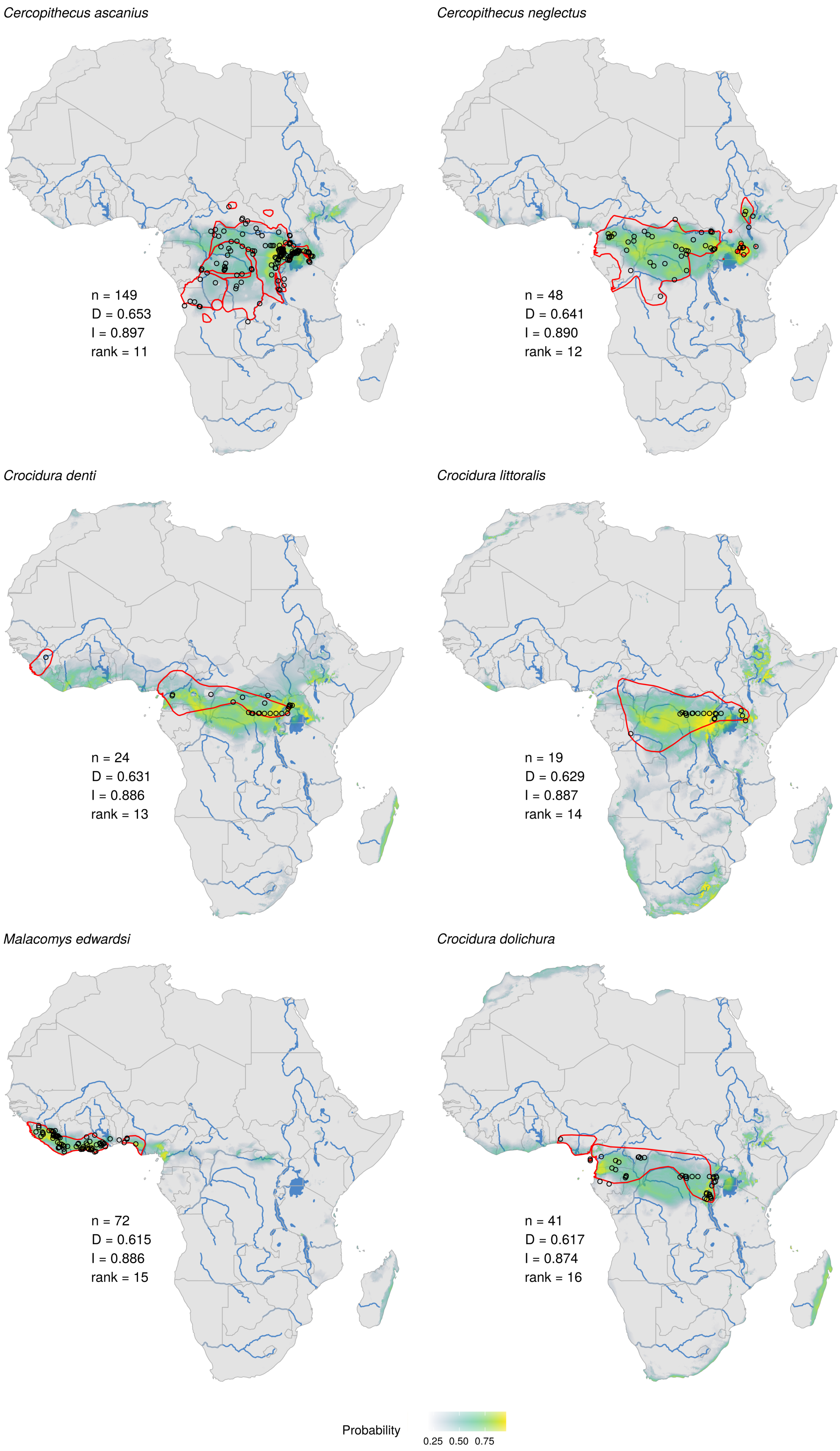
Cercopithecus ascanius
n = 149
D = 0.653
I = 0.897
rank = 11
Cercopithecus neglectus
n = 48
D = 0.641
I = 0.890
rank = 12
Crocidura denti
n = 24
D = 0.631
I = 0.886
rank = 13
Crocidura littoralis
n = 19
D = 0.629
I = 0.887
rank = 14
Malacomys edwardsi
n = 72
D = 0.615
I = 0.886
rank = 15
Crocidura dolichura
n = 41
D = 0.617
I = 0.874
rank = 16
Probability
0.25 0.50 0.75

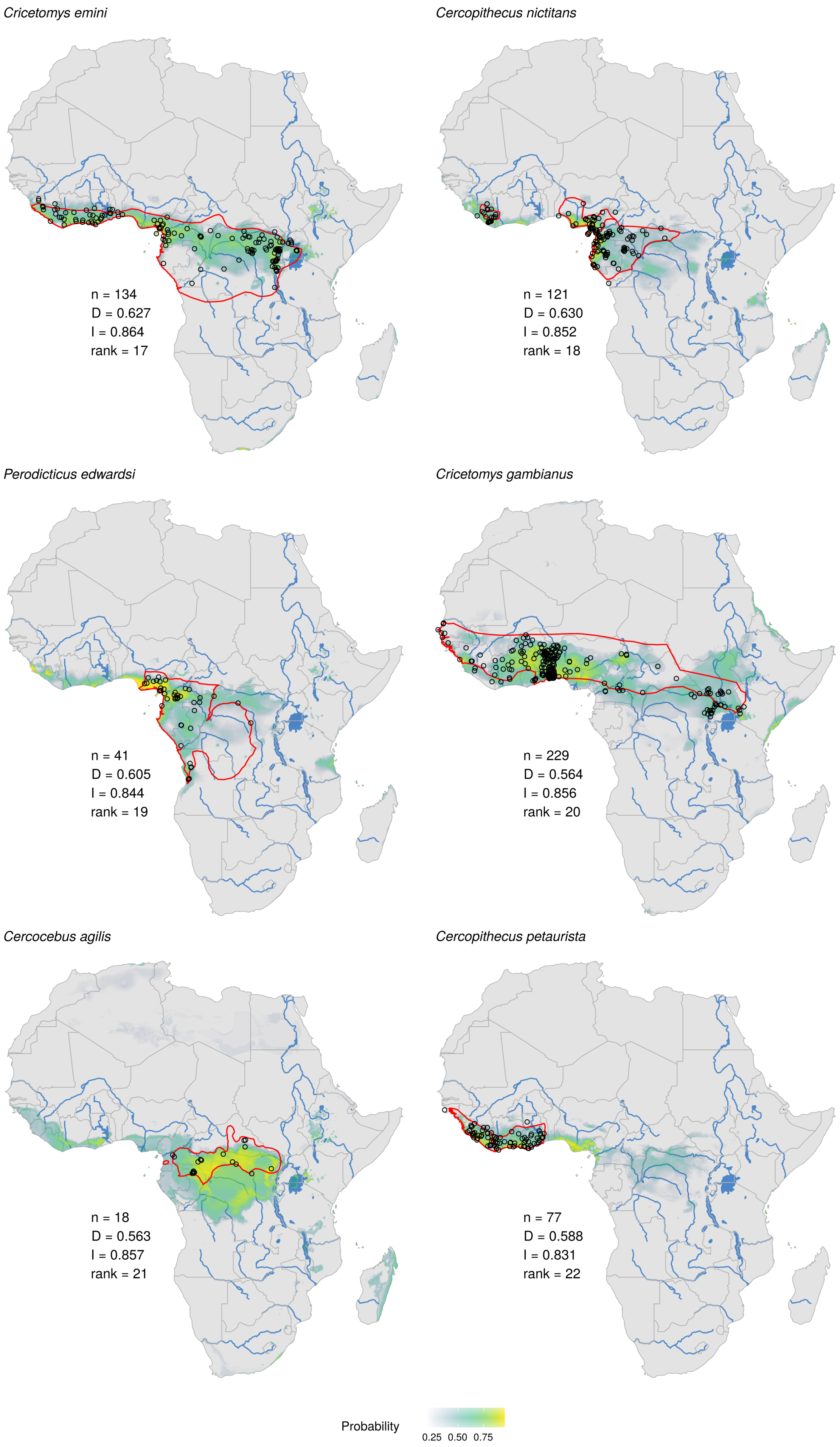
Cricetomys emini
n = 134
D = 0.627
I = 0.864
rank = 17
Cercopithecus nictitans
n = 121
D = 0.630
I = 0.852
rank = 18
Perodicticus edwardsi
n = 41
D = 0.605
I = 0.844
rank = 19
Cricetomys gambianus
n = 229
D = 0.564
I = 0.856
rank = 20
Cercocebus agilis
n = 18
D = 0.563
I = 0.857
rank = 21
Cercopithecus petaurista
n = 77
D = 0.588
I = 0.831
rank = 22
Probability
0.25 0.50 0.75

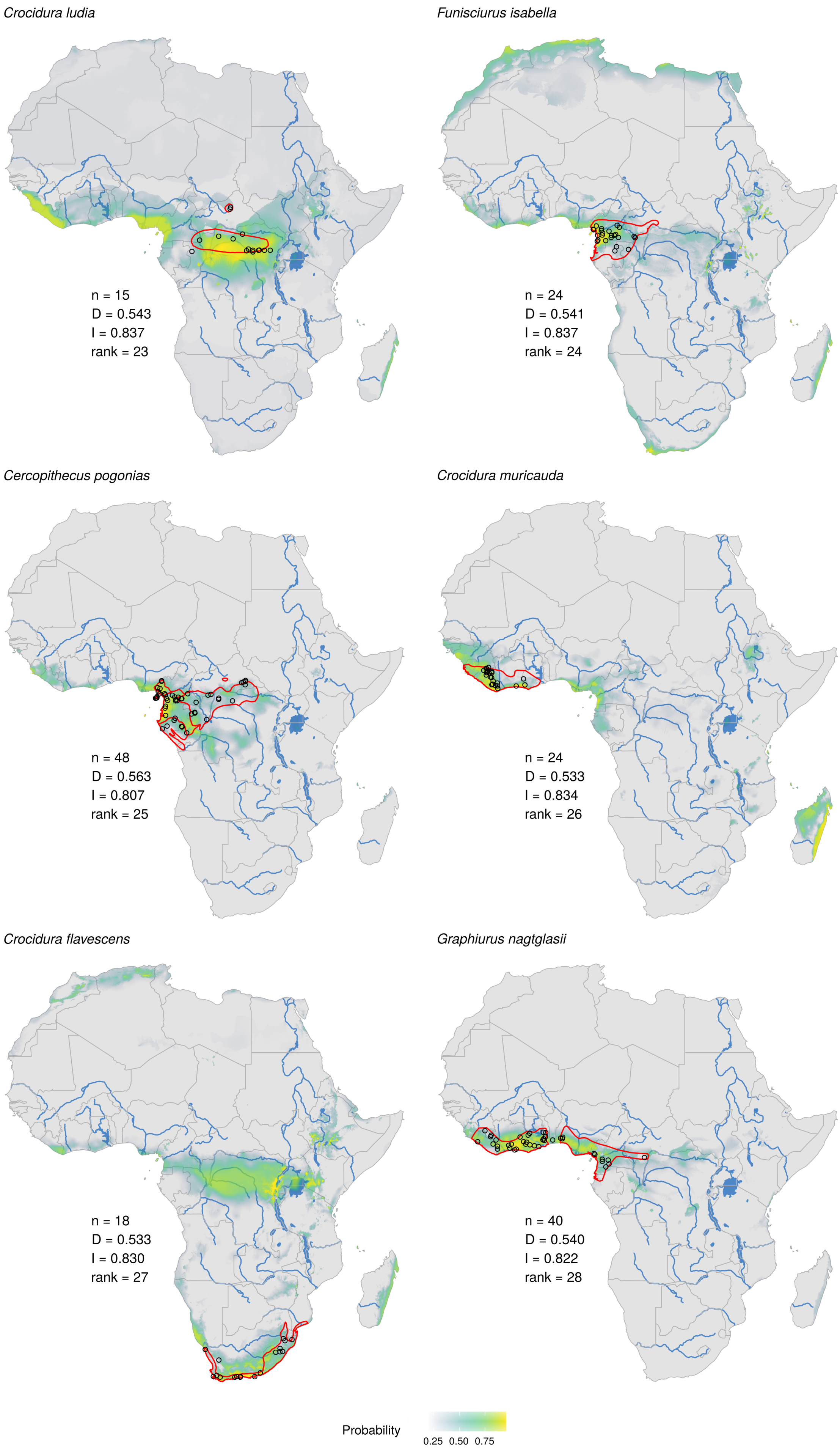
Crocidura ludia
n = 15
D = 0.543
I = 0.837
rank = 23
Funisciurus isabella
n = 24
D = 0.541
I = 0.837
rank = 24
Cercopithecus pogonias
n = 48
D = 0.563
I = 0.807
rank = 25
Crocidura muricauda
n = 24
D = 0.533
I = 0.834
rank = 26
Crocidura flavescens
n = 18
D = 0.533
I = 0.830
rank = 27
Graphiurus nagtglasii
n = 40
D = 0.540
I = 0.822
rank = 28
Probability
0.25 0.50 0.75

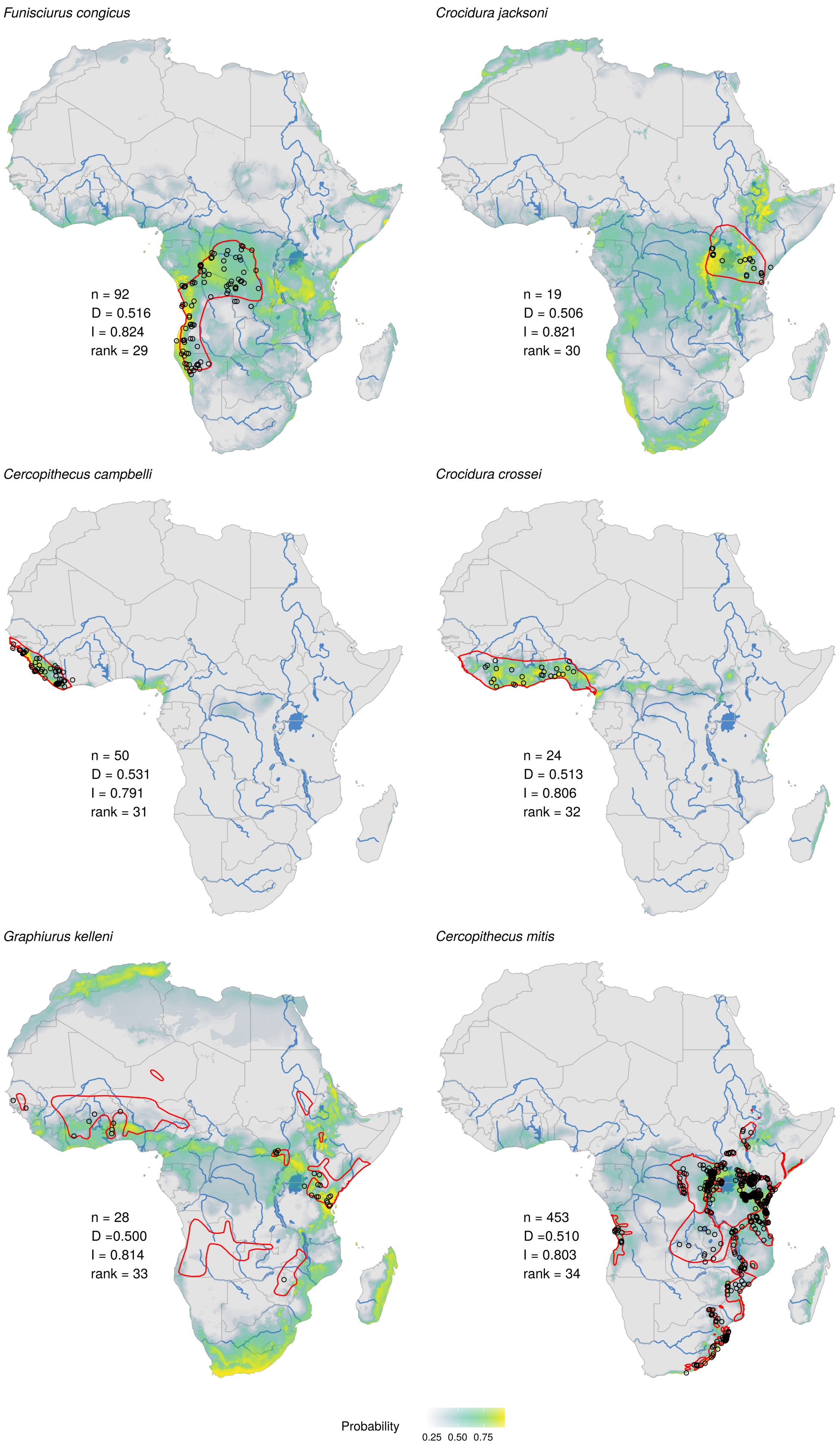
Funisciurus congicus
n = 92
D = 0.516
I = 0.824
rank = 29
Crocidura jacksoni
n = 19
D = 0.506
I = 0.821
rank = 30
Cercopithecus campbelli
n = 50
D = 0.531
I = 0.791
rank = 31
Crocidura crossei
n = 24
D = 0.513
I = 0.806
rank = 32
Graphiurus kelleni
n = 28
D =0.500
I = 0.814
rank = 33
Cercopithecus mitis
n = 453
D =0.510
I = 0.803
rank = 34
Probability
0.25 0.50 0.75

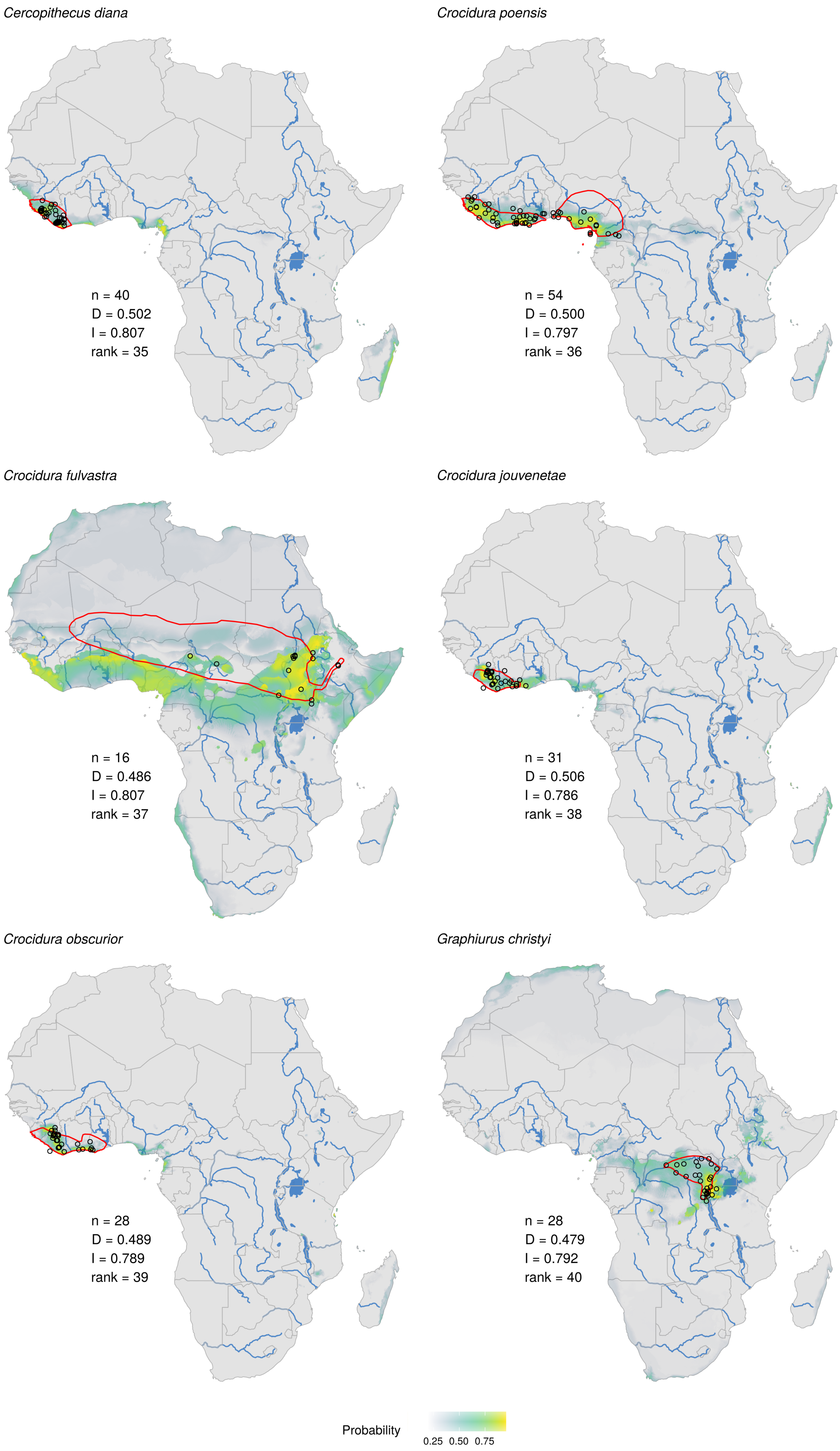
Cercopithecus diana
n = 40
D = 0.502
I = 0.807
rank = 35
Crocidura poensis
n = 54
D = 0.500
I = 0.797
rank = 36
Crocidura fulvastra
n = 16
D = 0.486
I = 0.807
rank = 37
Crocidura jouvenetae
n = 31
D = 0.506
I = 0.786
rank = 38
Crocidura obscurior
n = 28
D = 0.489
I = 0.789
rank = 39
Graphiurus christyi
n = 28
D = 0.479
I = 0.792
rank = 40
Probability
0.25 0.50 0.75

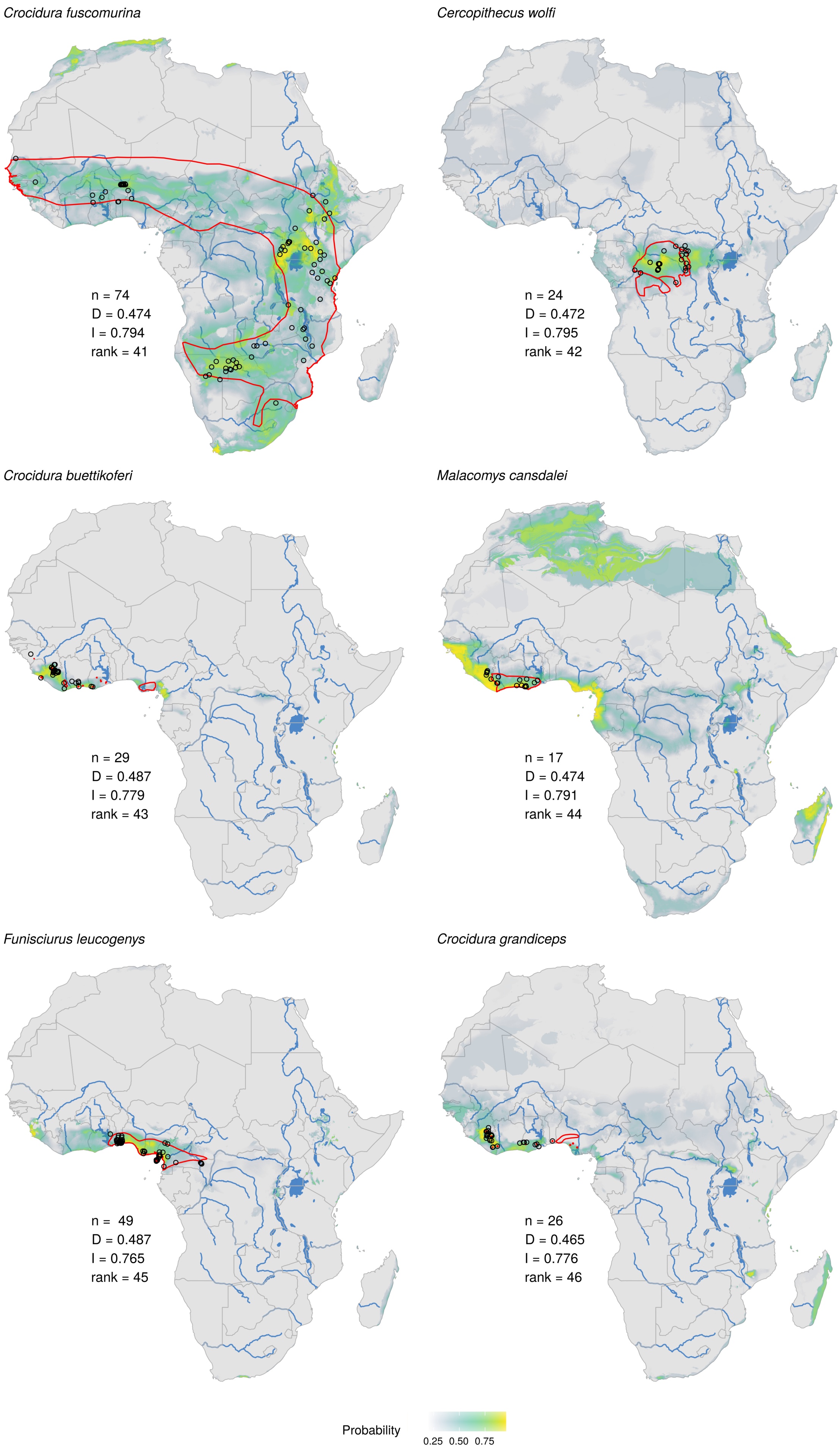

Crocidura fuscomurina
n = 74
D = 0.474
I = 0.794
rank = 41
Cercopithecus wolfi
n = 24
D = 0.472
I = 0.795
rank = 42
Crocidura buettikoferi
n = 29
D = 0.487
I = 0.779
rank = 43
Malacomys cansdalei
n = 17
D = 0.474
I = 0.791
rank = 44
Funisciurus leucogenys
n = 49
D = 0.487
I = 0.765
rank = 45
Crocidura grandiceps
n = 26
D = 0.465
I = 0.776
rank = 46
Probability
0.25 0.50 0.75

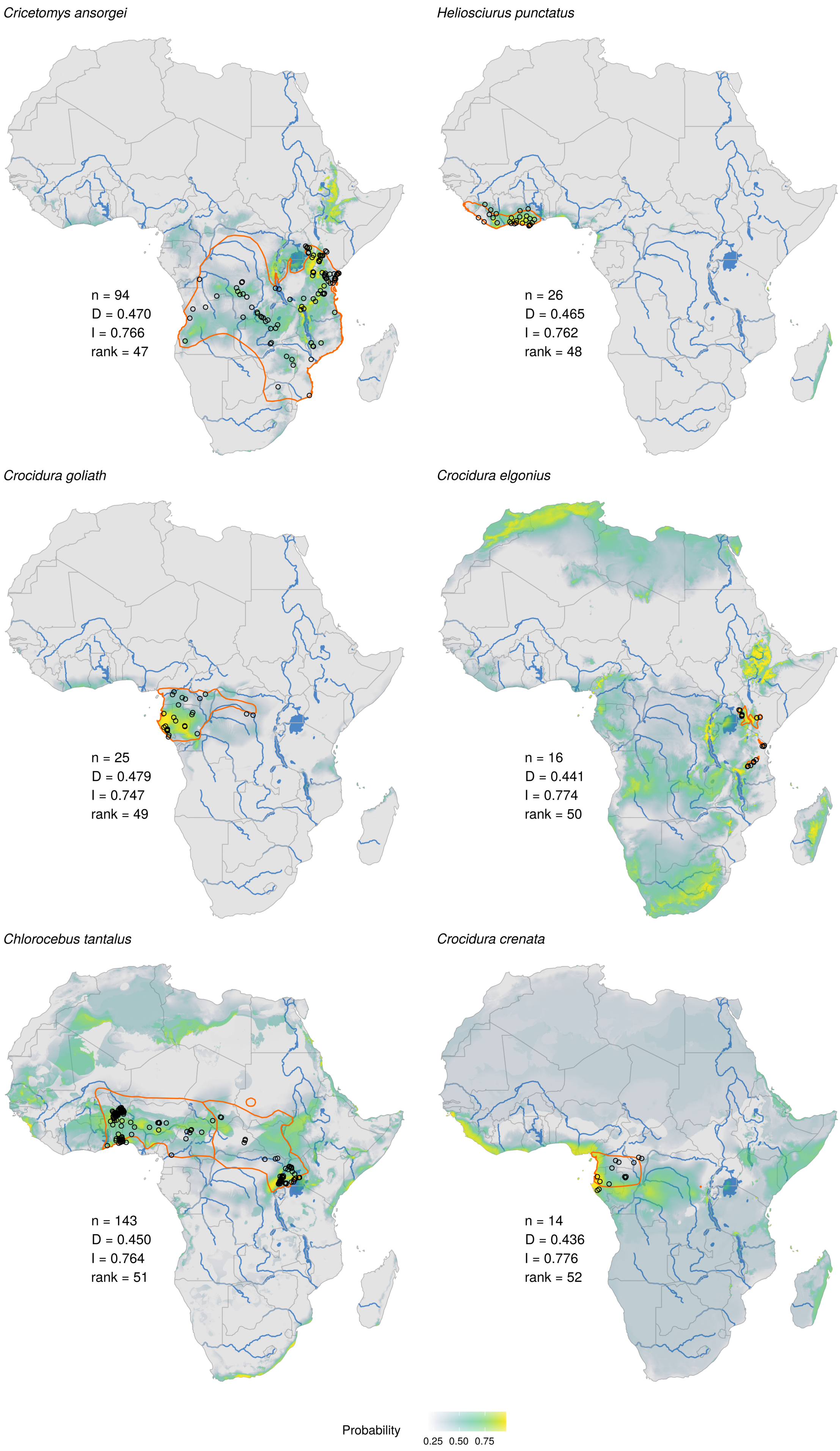
Cricetomys ansorgei
n = 94
D = 0.470
I = 0.766
rank = 47
Heliosciurus punctatus
n = 26
D = 0.465
I = 0.762
rank = 48
Crocidura goliath
n = 25
D = 0.479
I = 0.747
rank = 49
Crocidura elgonius
n = 16
D = 0.441
I = 0.774
rank = 50
Chlorocebus tantalus
n = 143
D = 0.450
I = 0.764
rank = 51
Crocidura crenata
n = 14
D = 0.436
I = 0.776
rank = 52
Probability
0.25 0.50 0.75

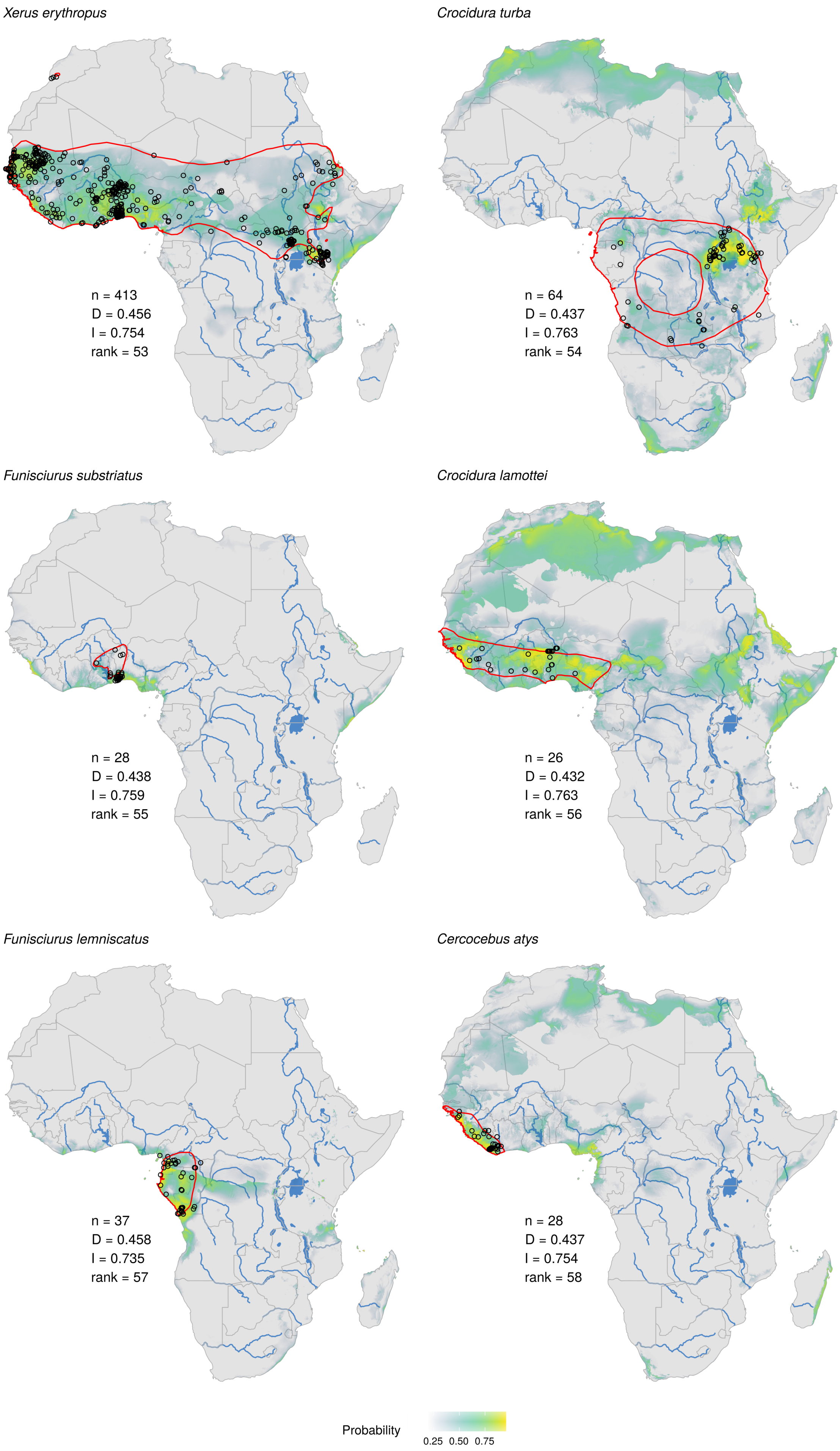
Xerus erythropus
n = 413
D = 0.456
I = 0.754
rank = 53
Crocidura turba
n = 64
D = 0.437
I = 0.763
rank = 54
Funisciurus substriatus
n = 28
D = 0.438
I = 0.759
rank = 55
Crocidura lamottei
n = 26
D = 0.432
I = 0.763
rank = 56
Funisciurus lemniscatus
n = 37
D = 0.458
I = 0.735
rank = 57
Cercocebus atys
n = 28
D = 0.437
I = 0.754
rank = 58
Probability
0.25 0.50 0.75

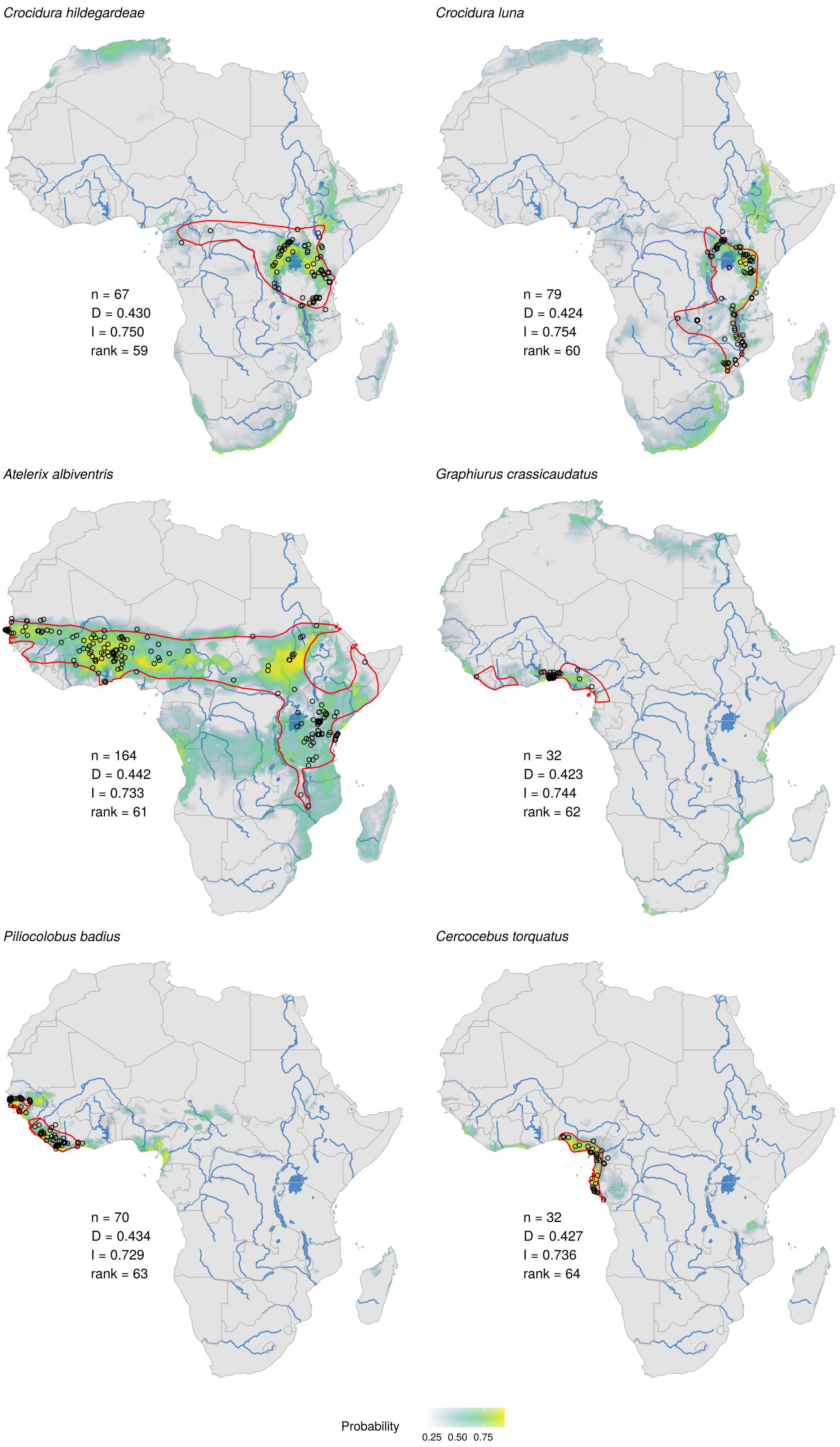
Crocidura hildegardeae
n = 67
D = 0.430
I = 0.750
rank = 59
Crocidura luna
n = 79
D = 0.424
I = 0.754
rank = 60
Atelerix albiventris
n = 164
D = 0.442
I = 0.733
rank = 61
Graphiurus crassicaudatus
n = 32
D = 0.423
I = 0.744
rank = 62
Piliocolobus badius
n = 70
D = 0.434
I = 0.729
rank = 63
Cercocebus torquatus
n = 32
D = 0.427
I = 0.736
rank = 64
Probability
0.25 0.50 0.75

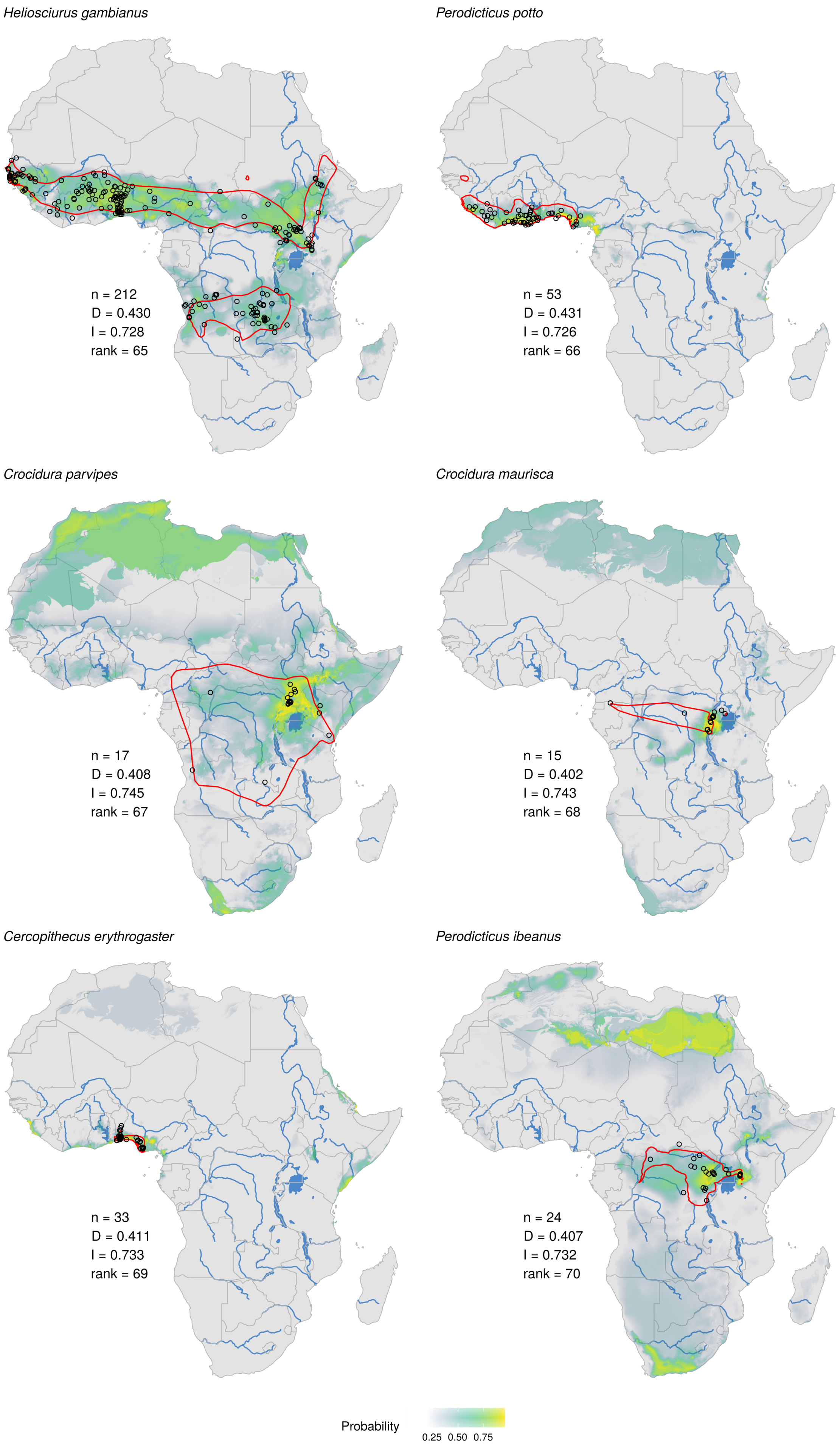
Heliosciurus gambianus
n = 212
D = 0.430
I = 0.728
rank = 65
Perodicticus potto
n = 53
D = 0.431
I = 0.726
rank = 66
Crocidura parvipes
n = 17
D = 0.408
I = 0.745
rank = 67
Crocidura maurisca
n = 15
D = 0.402
I = 0.743
rank = 68
Cercopithecus erythrogaster
n = 33
D = 0.411
I = 0.733
rank = 69
Perodicticus ibeanus
n = 24
D = 0.407
I = 0.732
rank = 70
Probability
0.25 0.50 0.75

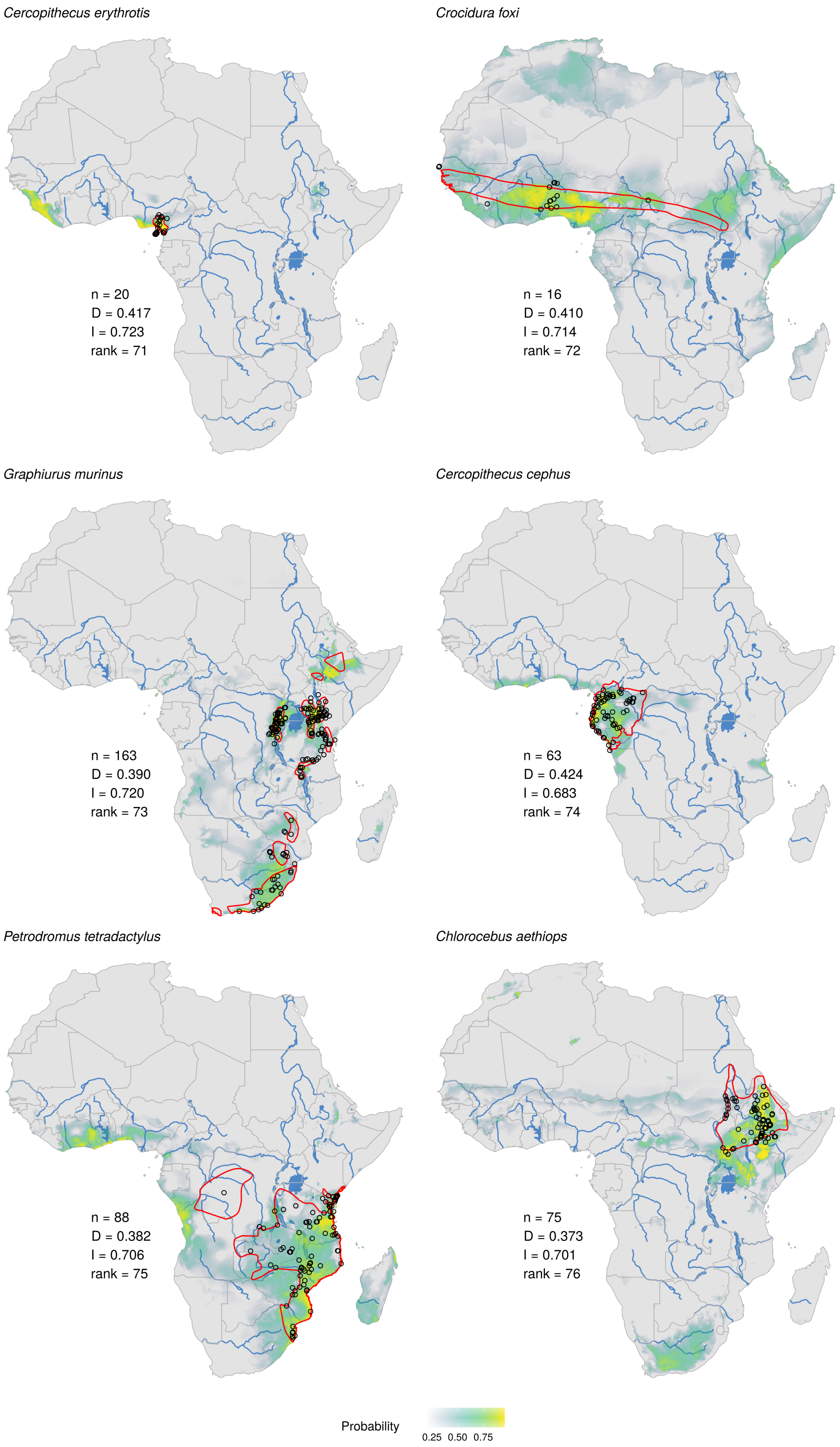
Cercopithecus erythrotis
n = 20
D = 0.417
I = 0.723
rank = 71
Crocidura foxi
n = 16
D = 0.410
I = 0.714
rank = 72
Graphiurus murinus
n = 163
D = 0.390
I = 0.720
rank = 73
Cercopithecus cephus
n = 63
D = 0.424
I = 0.683
rank = 74
Petrodromus tetradactylus
n = 88
D = 0.382
I = 0.706
rank = 75
Chlorocebus aethiops
n = 75
D = 0.373
I = 0.701
rank = 76
Probability
0.25 0.50 0.75

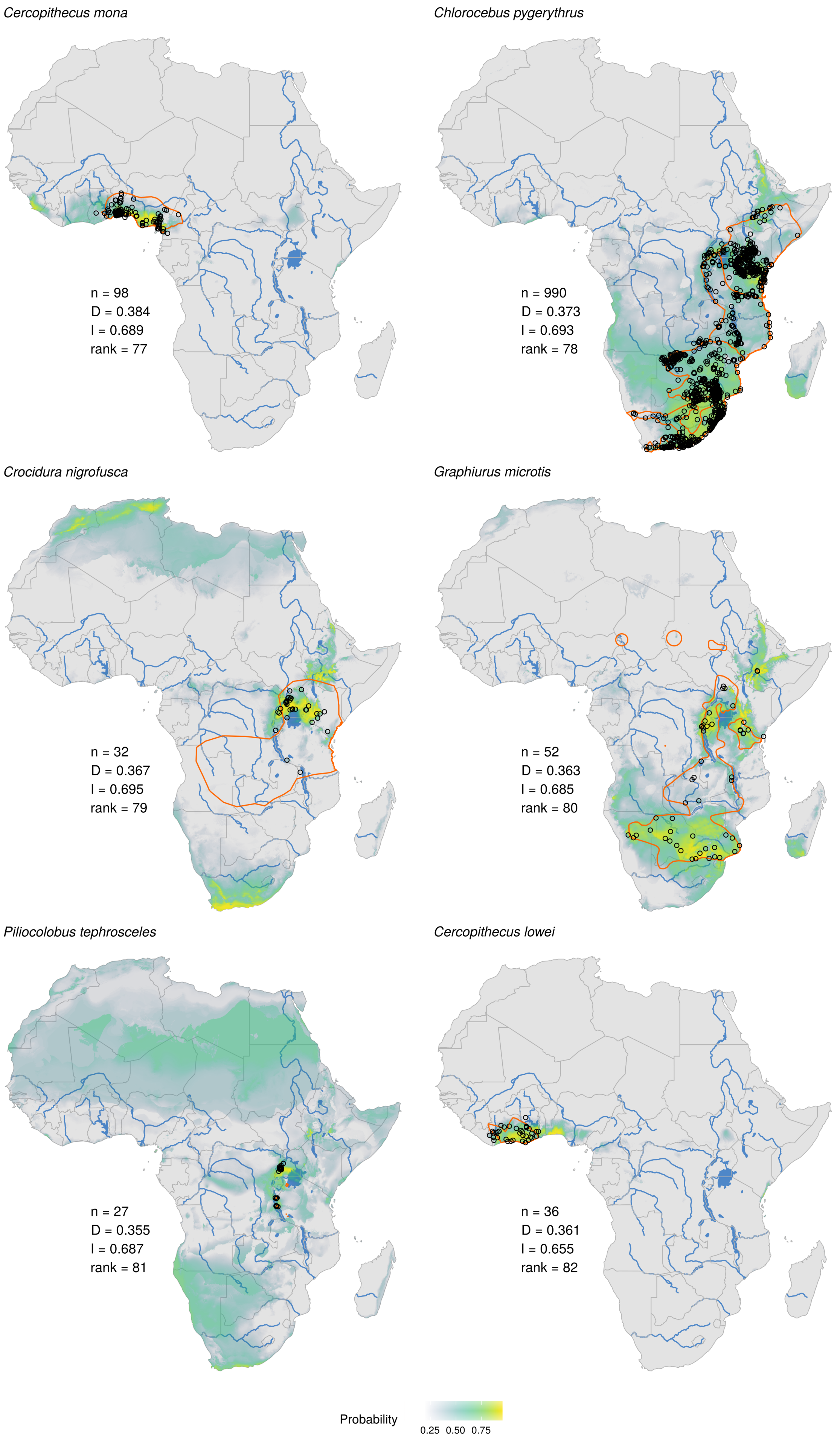
Cercopithecus mona
n = 98
D = 0.384
I = 0.689
rank = 77
Chlorocebus pygerythrus
n = 990
D = 0.373
I = 0.693
rank = 78
Crocidura nigrofusca
n = 32
D = 0.367
I = 0.695
rank = 79
Graphiurus microtis
n = 52
D = 0.363
I = 0.685
rank = 80
Piliocolobus tephrosceles
n = 27
D = 0.355
I = 0.687
rank = 81
Cercopithecus lowei
n = 36
D = 0.361
I = 0.655
rank = 82
Probability
0.25 0.50 0.75

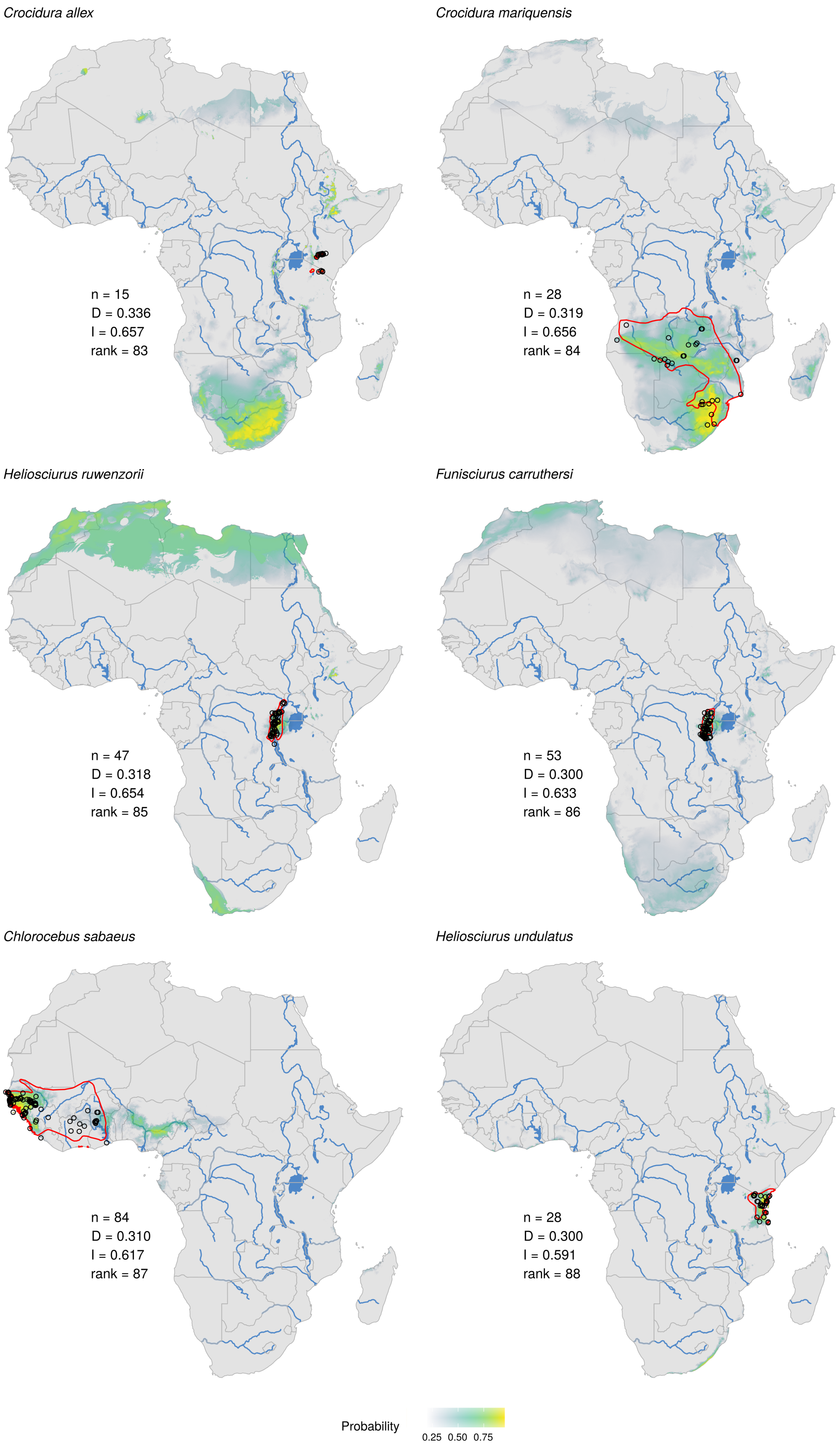

Crocidura allex
n = 15
D = 0.336
I = 0.657
rank = 83
Crocidura mariquensis
n = 28
D = 0.319
I = 0.656
rank = 84
Heliosciurus ruwenzorii
n = 47
D = 0.318
I = 0.654
rank = 85
Funisciurus carruthersi
n = 53
D = 0.300
I = 0.633
rank = 86
Chlorocebus sabaeus
n = 84
D = 0.310
I = 0.617
rank = 87
Heliosciurus undulatus
n = 28
D = 0.300
I = 0.591
rank = 88
Probability
0.25 0.50 0.75

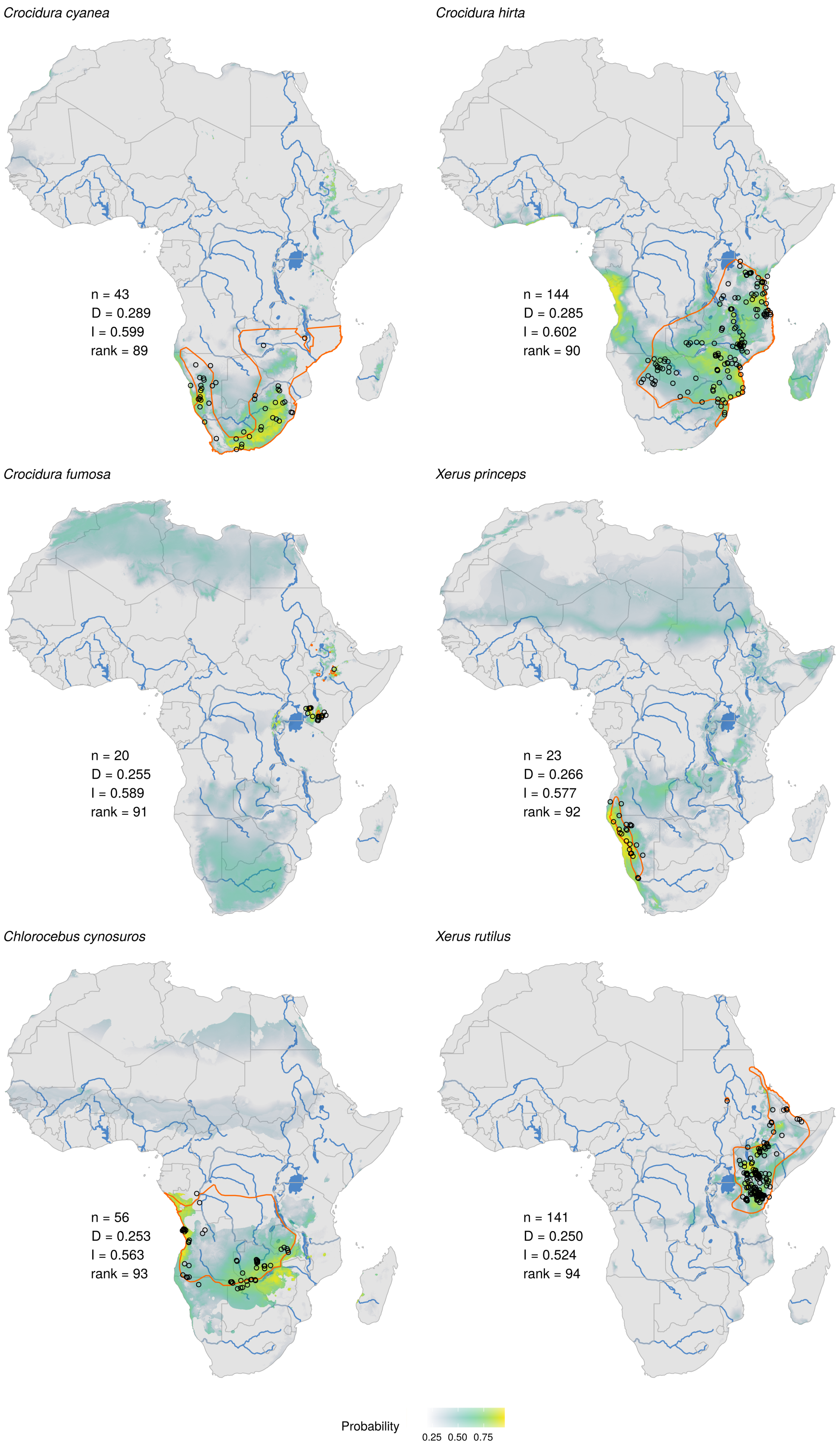
Crocidura cyanea
n = 43
D = 0.289
I = 0.599
rank = 89
Crocidura hirta
n = 144
D = 0.285
I = 0.602
rank = 90
Crocidura fumosa
n = 20
D = 0.255
I = 0.589
rank = 91
Xerus princeps
n = 23
D = 0.266
I = 0.577
rank = 92
Chlorocebus cynosuros
n = 56
D = 0.253
I = 0.563
rank = 93
Xerus rutilus
n = 141
D = 0.250
I = 0.524
rank = 94
Probability
0.25 0.50 0.75

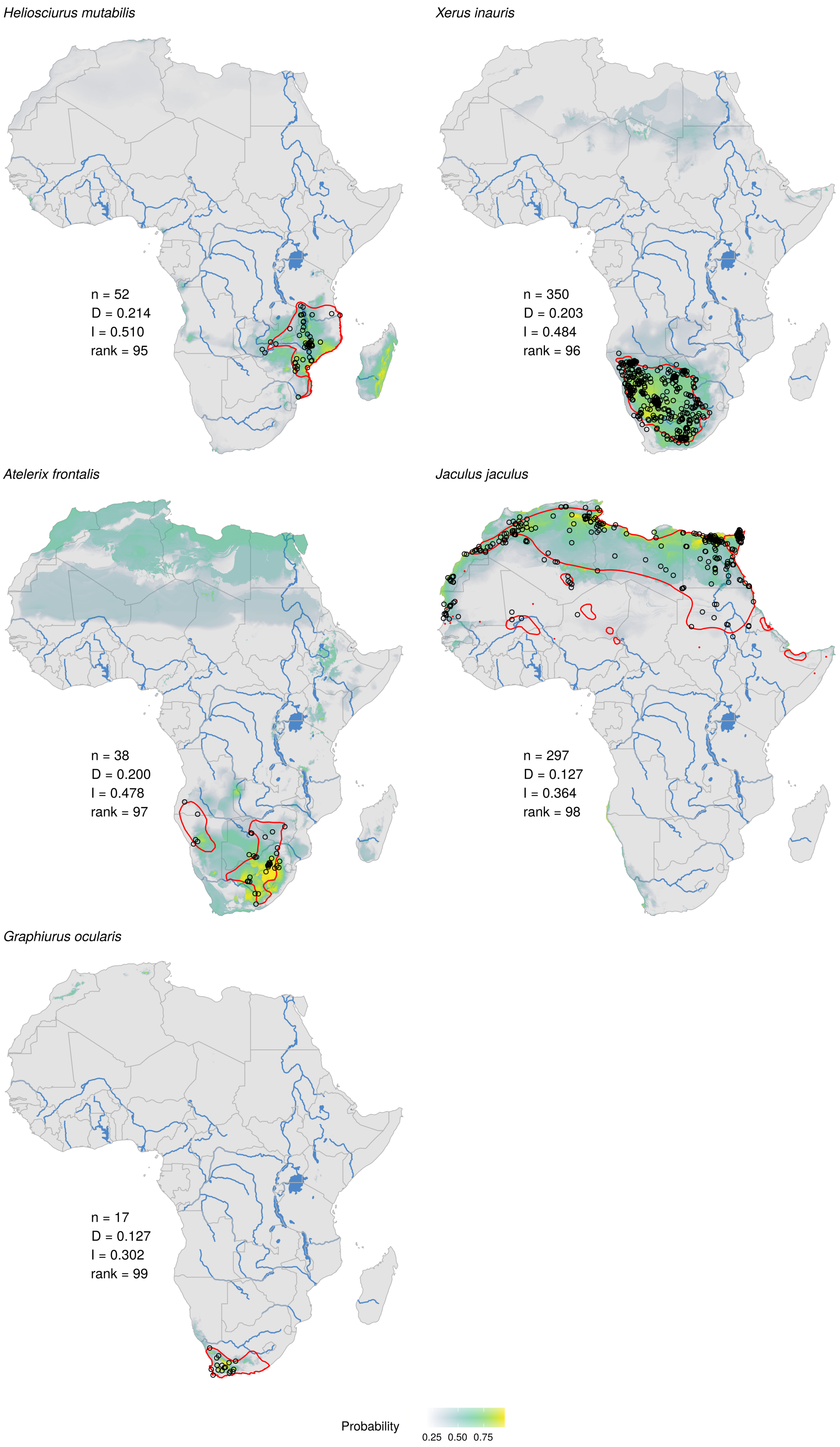
Heliosciurus mutabilis
n = 52
D = 0.214
I = 0.510
rank = 95
Xerus inauris
n = 350
D = 0.203
I = 0.484
rank = 96
Atelerix frontalis
n = 38
D = 0.200
I = 0.478
rank = 97
Jaculus jaculus
n = 297
D = 0.127
I = 0.364
rank = 98
Graphiurus ocularis
n = 17
D = 0.127
I = 0.302
rank = 99
Probability
0.25 0.50 0.75

Figure S2: Standard deviation calculated for the ecological niche of MPXV and that of the 10 mammal species showing the best overlap with the MPXV niche.

Black circles indicate localities used to build the distribution model. The probabilities of occurrence are highlighted using different colours: blue grey for standard deviation < 0.2; turquoise green for 0.2 < sd < 0.3; yellowish green for 0.3 < sd < 0.35; and yellow for sd > 0.4. The red line is the IUCN distribution of the species [60]. At the left of the maps are indicated the number of occurrence records (n) used to infer the ecological niche, the Schoener's D and Hellinger's I values summarizing niche overlap between mammal species and MPXV, and the rank based on overlap analyses with the MPXV niche.

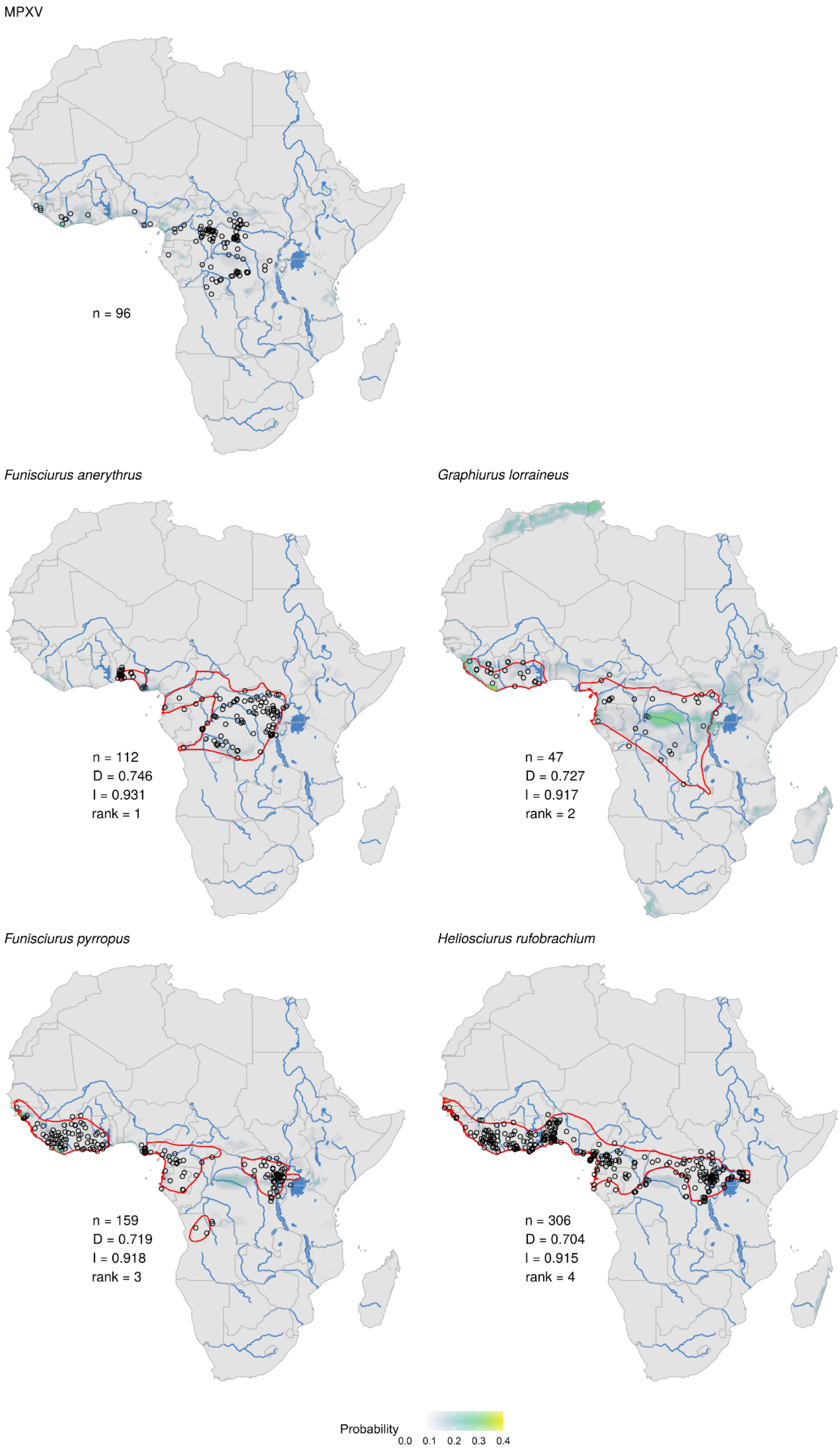
MPXV
n = 96
Funisciurus anerythrus
n = 112
D = 0.746
I = 0.931
rank = 1
Graphiurus lorraineus
n = 47
D = 0.727
I = 0.917
rank = 2
Funisciurus pyrropus
n = 159
D = 0.719
I = 0.918
rank = 3
Heliosciurus rufobrachium
n = 306
D = 0.704
I = 0.915
rank = 4
Probability
0.0
0.1
0.2
0.3
0.4

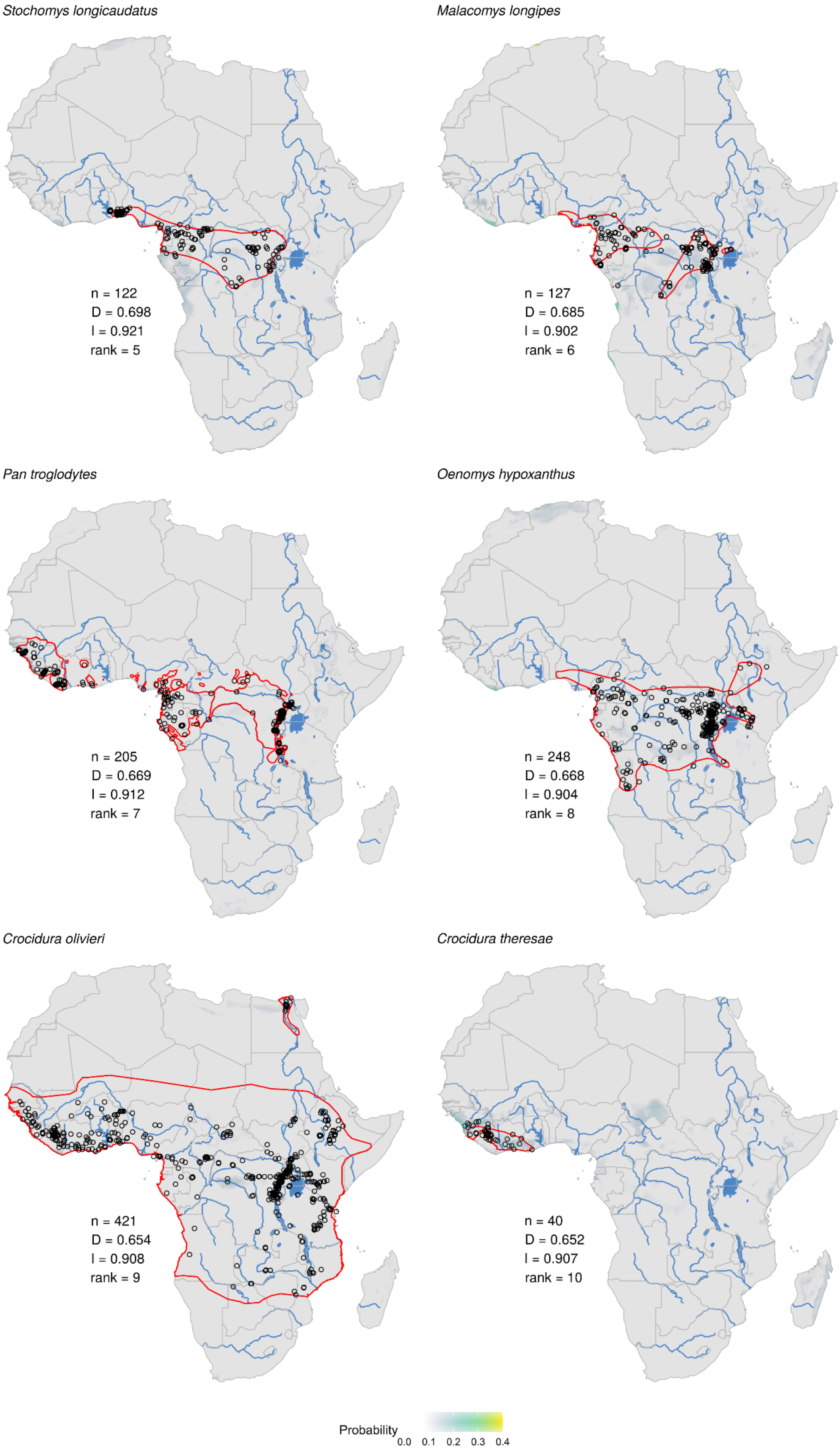
Stochomys longicaudatus
n = 122
D = 0.698
I = 0.921
rank = 5
Malacomys longipes
n = 127
D = 0.685
I = 0.902
rank = 6
Pan troglodytes
n = 205
D = 0.669
I = 0.912
rank = 7
Oenomys hypoxanthus
n = 248
D = 0.668
I = 0.904
rank = 8
Crocidura olivieri
n = 421
D = 0.654
I = 0.908
rank = 9
Crocidura theresae
n = 40
D = 0.652
I = 0.907
rank = 10
Probability
0.0 0.1 0.2 0.3 0.4
